# Extracellular acidosis restricts one-carbon metabolism and preserves T cell stemness

**DOI:** 10.1038/s42255-022-00730-6

**Published:** 2023-01-30

**Authors:** Hongcheng Cheng, Yajing Qiu, Yue Xu, Li Chen, Kaili Ma, Mengyuan Tao, Luke Frankiw, Hongli Yin, Ermei Xie, Xiaoli Pan, Jing Du, Zhe Wang, Wenjie Zhu, Lu Chen, Lianjun Zhang, Guideng Li

**Affiliations:** 1 Key Laboratory of Synthetic Biology Regulatory Element, Institute of Systems Medicine, Chinese Academy of Medical Sciences and Peking Union Medical College, Beijing, China; 2 Suzhou Institute of Systems Medicine, Suzhou, China; 3 Key Laboratory of Birth Defects and Related Diseases of Women and Children of MOE, Department of Laboratory Medicine, State Key Laboratory of Biotherapy, West China Second Hospital, Sichuan University, Chengdu, Sichuan China; 4 School of Life Science and Technology, China Pharmaceutical University, Nanjing, China; 5 Department of Pediatrics, Boston Children’s Hospital, Boston, MA USA; 6 Institute of Pharmaceutical Sciences, China Pharmaceutical University, Nanjing, China; 7 Jiangsu Center for the Collaboration and Innovation of Cancer Biotherapy, Cancer Institute, Xuzhou Medical University, Xuzhou, China

**Keywords:** Tumour immunology, Immunotherapy, Metabolism, Cytotoxic T cells

## Abstract

The accumulation of acidic metabolic waste products within the tumor microenvironment inhibits effector functions of tumor-infiltrating lymphocytes (TILs). However, it remains unclear how an acidic environment affects T cell metabolism and differentiation. Here we show that prolonged exposure to acid reprograms T cell intracellular metabolism and mitochondrial fitness and preserves T cell stemness. Mechanistically, elevated extracellular acidosis impairs methionine uptake and metabolism via downregulation of SLC7A5, therefore altering H3K27me3 deposition at the promoters of key T cell stemness genes. These changes promote the maintenance of a ‘stem-like memory’ state and improve long-term in vivo persistence and anti-tumor efficacy in mice. Our findings not only reveal an unexpected capacity of extracellular acidosis to maintain the stem-like properties of T cells, but also advance our understanding of how methionine metabolism affects T cell stemness.

## Abstract (web summary)

Prolonged exposure to acid is shown to reprogram T cell intracellular methionine metabolism to preserve T cell stemness and increase anti-tumor efficacy.

## Main

Adoptive transfer of tumor-antigen-specific T cells represents a major advance in the field of cancer therapy, as it has led to the complete regression of certain malignancies. Its therapeutic efficacy relies heavily on the persistence and differentiation status of the transferred T cells^1,2^. Less differentiated memory T cells are the preferred population for adoptive T cell transfer (ACT) due to their stem-cell-like properties of self-renewal and multipotency^3,4^. Indeed, the use of stem-like memory T cells for ACT has been shown to achieve superior anti-tumor responses^5^.

As compared with terminally differentiated effector T cells, stem-like T cells display distinct hallmarks^6^. Both human and mouse stem-like T cells are characterized by their expression of antigen-experienced and homing-associated molecules, including high levels of CD62L and CCR7 (ref. ^7^). Our understanding of the transcriptional profiles, epigenetic modifications and metabolic pathways that regulate stem-like T cells has dramatically advanced in recent years^8–10^. Several transcriptional factors, such as TCF1, KLF2 and LEF1, have been reported to play essential roles in driving or maintaining T cell stemness^9^. Notably, TCF1 is the key transcription factor that promotes the generation of long-lived memory T cells^11^. During chronic infections, a small fraction of TCF1^+^ stem-like T cells sustain T cell responses against secondary infection^1,12^. Epigenetic changes provide a means for T cells to both initiate the transcriptional changes that underlie the acquisition of memory-cell characteristics and maintain these transcriptional expression patterns^13^. Multiple studies have demonstrated that trimethylation of histone H3 at K4 (H3K4me3), an activation-associated modification, is gained at memory-associated gene loci, including *TCF7*, *KLF2*, *LEF1*, *CCR7* and *SELL*, during the differentiation of naive CD8^+^ T cells into memory T cells, whereas trimethylation of H3 at K27 (H3K27me3), a repressive modification, is lost at these loci^13–15^. Conversely, effector-associated genes (*GZMB*, *PRF1*, *IFNG* and *TBX21*) demonstrate decreased repressive and increased activating epigenetic modifications at these loci in effector T cells^13,16^. Finally, accumulating lines of evidence suggest that metabolic circuits dictate T cell fate decisions and shape their epigenetic and functional states^17^. Short-lived effector T cells are highly glycolytic and dependent on one-carbon metabolism^18,19^, whereas stem-like memory T cells display distinct metabolic profiles characterized by increased fatty acid oxidation (FAO) and mitochondrial spare respiratory capacity (SRC), the cardinal characteristics involved in long-term persistence^20–22^. Therefore, metabolic reprogramming is important for the efficient acquisition of stemness and long-term survival of T cells.

The immunosuppressive tumor microenvironment (TME), characterized by low pH, hypoxia, glucose deprivation and lactic acid enrichment, is the key barrier hindering proper T cell expansion, differentiation and functionality^23,24^. Indeed, metabolic stress imposed by the TME impairs mitochondrial capacity and fitness, triggering intratumoral T cell metabolic insufficiency and dysfunction^25–28^. Intriguingly, most tumor-infiltrating lymphocytes (TILs) are dysfunctional, but a small fraction harbors stem-like memory or precursor properties^7,29^. This stem-like TIL subset preserves proliferative potential and persistence and is associated with a favorable response to immune checkpoint blockade (ICB) and TIL-ACT in people with cancer^11,30^. Previous studies have shown that elevated extracellular acidosis (↑[H^+^]) suppressed the cytolytic activity of T cells both in vitro and in vivo^31–33^. In addition, the acidic TME has a considerable influence on the activity and differentiation of tumor-infiltrating myeloid cells, such as dendritic cells (DCs) and tumor-associated macrophages (TAMs)^34–36^. However, the impact of ↑[H^+^] on T cell stemness and metabolic fitness remains largely unknown.

Here, we report that long-term in vitro ↑[H^+^] exposure facilitates the differentiation of human and mouse stem-like CD8^+^ T cells at the expense of terminal effector CD8^+^ T cells. We found that long-term ↑[H^+^] treatment remodels T cell metabolism and sustains mitochondrial respiratory capacity. Further, persistent ↑[H^+^] exposure impairs methionine uptake and metabolism, which subsequently results in decreased H3K27me3 deposition of memory-related genes, thereby facilitating the maintenance of a ‘stem-like memory’ status. Finally, adoptive transfer of T cells cultured in ↑[H^+^]-conditions shows potent anti-tumor activity in vivo, in line with their less exhausted phenotype. Thus, our study reveals the unexpected role of extracellular acidosis in preserving T cell stemness via remodeling of cellular metabolism and epigenetic patterns.

## Results

### ↑[H^+^] exposure promotes CD8^+^ T cell stemness

**Fig. 1:**
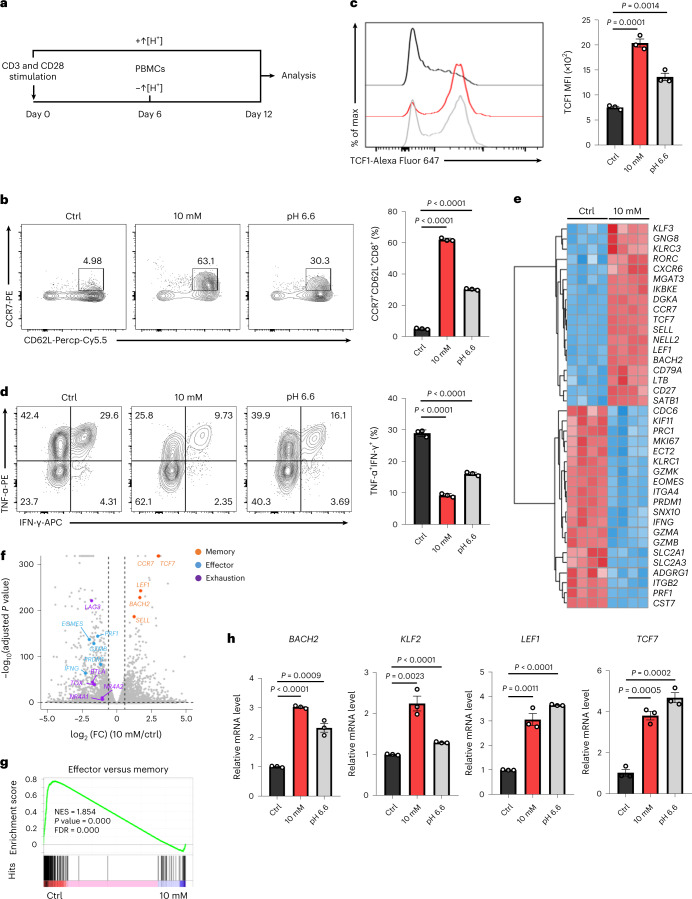
↑[H^+^] exposure facilitates the differentiation of stem-like CD8^+^ T cells. **a**, Schematic of human T cell activation in the indicated conditions: pH 7.4 (–↑[H^+^], control), pH 6.6 (+↑[H^+^], hydrochloric acid), or 10 mM lactic acid (+↑[H^+^]). PBMCs, peripheral blood mononuclear cells. **b**, Representative CCR7 and CD62L expression profiles in human CD8^+^ T cells under different conditions at day 12. *n* = 3 independent samples. **c**, Representative histograms and quantification of TCF1 expression in human CD8^+^ T cells under different conditions at day 12. *n* = 3 independent samples. MFI, mean fluorescence intensity. **d**, Human T cells were expanded as in **a** for 12 days and stimulated with phorbol 12-myristate 13-acetate (PMA) containing brefeldin A (BFA) for 4.5 h. The intracellular expression profile of IFN-γ and TNF-α is depicted for T cells in the pH 7.4 (left), 10 mM lactic acid (middle) or pH 6.6 (right) condition. *n* = 3 independent samples. **e**,**f**, RNA-seq analysis of human T cells that were expanded in control (pH 7.4) or lactic acid (10 mM). Heat map of selected genes (**e**) and volcano plot of all genes in which genes associated with memory, effector and exhausted T cells were labeled (**f**). In the volcano plot, the *x* axis represents the log_2_-transformed fold change (FC) values for cells treated with lactic acid relative to controls at day 12, and the *y* axis represents the adjusted *P* values. *n* = 4 independent samples. **g**, GSEA plot comparing control with lactic-acid-conditioned T cells for effector versus memory enrichment. NES, normalized enrichment score. **h**, Quantitative mRNA expression of transcription factors associated with T cell stemness (*BACH2, KLF2*, *LEF1*, *TCF7*) in T cells under the indicated conditions. *n* = 3 independent samples. Data are presented as mean ± s.e.m. Statistical analyses were determined by unpaired two-tailed Student’s *t*-test (**b**–**d**,**h**). Nominal *P* values and false-discovery rates (FDRs) were calculated with default method of the GSEA software (**g**). Source data

**Extended Data Fig. 1:**
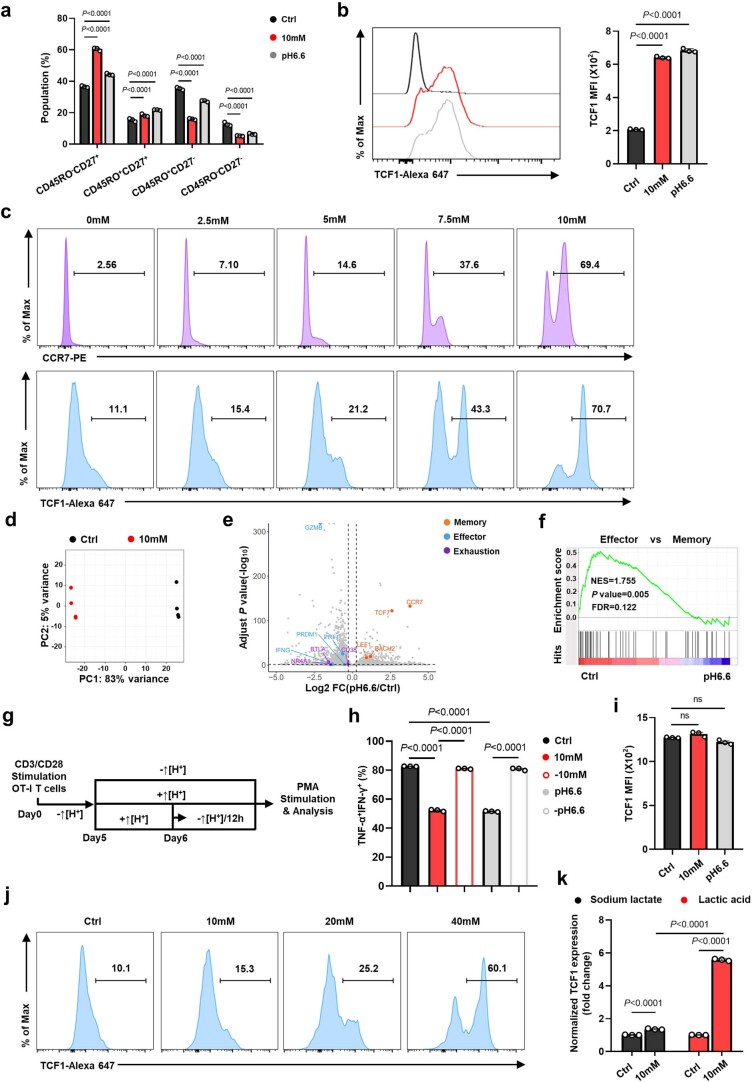
Elevated [H^+^] promotes T cell differentiation to memory state. **(a)** Representative FACS plots of CD45RO/CD27 human CD8^+^ T cells under different conditions at day 12. n = 3 independent samples. (**b**) Representative histograms of TCF1 expression in OT-I CD8^+^ T cells cultured in indicated conditions for 6 days. n = 3 independent samples. (**c**) Expression of CCR7 (purple) and TCF1 (blue) in human CD8^+^ T cells that were activated and expanded in indicated concentration of lactic acid medium for 12 days. (**d**) Principal component analysis (PCA) of RNA-seq data from control and lactic acid conditioned human T cells. (**e**) RNA-seq analysis of control (pH 7.4) and hydrochloric acid (pH 6.6) expanded human T cells. Differentially expressed genes associated with memory, effector, and exhaustion of T cells are depicted. Volcano plot with the x-axis representing the log_2_-transformed fold changes for cells treated with pH 6.6 (hydrochloric acid) relative to control at day 12 and y-axis representing the adjusted *P* values. (**f**) GSEA plots comparing control and hydrochloric acid conditioning T cells for effector versus memory enrichment. (**g,h**) OT-I CD8^+^ T cells were expanded in control medium for 5 days and transferred in pH 6.6 or 10 mM lactic acid conditions for 24 h, and then cultured within the indicated conditions for 12 h and stimulated with PMA containing BFA for 4.5 h (**g**). The percentages of IFN-γ^+^TNF-α^+^ population under different conditions are shown (**h**). n = 3 independent samples. (**i**) OT-I CD8^+^ T cells were expanded in control medium for 5 days and transferred in pH 6.6 or 10 mM lactic acid conditions from day 5–6 and then detected the expression of TCF1. n = 3 independent samples. (**j**) Expression of TCF1 in human CD8^+^ T cells that were activated and expanded at indicated concentration of sodium lactate medium for 12 days. (**k**) Statistical analysis of TCF1 expression in human CD8^+^ T cells conditioned with either 10 mM of lactic acid (red) or 10 mM of sodium lactate (black). n = 3 independent samples. Data are presented as mean ± SEM. Statistical analyses were determined by two-way ANOVA with Dunnett’s multiple-comparisons test (**a**), unpaired two-tailed Student’s *t*-test (**b,i**) or two-way ANOVA with Tukey’s multiple-comparisons test (**h,k**). Nominal *P* value and FDR were calculated with default method of GSEA software (**f**). ns, non-significant. Source data

To determine whether extracellular ↑[H^+^] affects stem-like T cell differentiation, we performed flow cytometric analysis of key stem-like phenotypes on human T cells cultured for 12 days in either control medium, ↑[H^+^] media containing 10 mM lactic acid (mimicking the lactate concentration in pathophysiological situations^23,35^) or acidic medium (~pH 6.6, by hydrochloric acid) (Fig. 1a). A higher proportion of stem-like CD8^+^ T cells, including early memory (CD45RO^−^CD27^+^) and central memory (CD45RO^+^CD27^+^), was seen in T cells conditioned in ↑[H^+^] versus control medium (Extended Data Fig. 1a). Additionally, we found that T cells cultured in ↑[H^+^] medium had a higher percentage of stem-like cells (CCR7^+^CD62L^+^) (Fig. 1b). Of note, we noticed markedly increased TCF1 expression, the key factor driving stem-like and central memory differentiation, at the protein level in both human and mouse CD8^+^ T cells exposed to ↑[H^+^] (Fig. 1c and Extended Data Fig. 1b). In addition, there was a [H^+^]-concentration-dependent effect on CCR7 and TCF1 expression (Extended Data Fig. 1c). In accordance with the stem-like phenotype of ↑[H^+^]-conditioned T cells, the production of intracellular interferon-γ (IFN-γ) and tumor necrosis factor α (TNF-α) was significantly reduced in these cells (Fig. 1d).

To better probe the T cell differentiation status, we performed RNA sequencing (RNA-seq) analysis and found that long-term in vitro ↑[H^+^] exposure resulted in a distinct transcriptional profile (Extended Data Fig. 1d). Exposure to ↑[H^+^] led to remarkably decreased expression of genes encoding effector molecules, such as *PRF1*, *GZMB* and *IFNG*, and the co-inhibitory receptor *BTLA* (Fig. 1e,f and Extended Data Fig. 1e). By contrast, ↑[H^+^]-cultured T cells demonstrated higher expression of *BACH2*, *CCR7*, *LEF1* and *TCF7*, which correlate with T cell stemness (Fig. 1e,f and Extended Data Fig. 1e). Gene set enrichment analysis (GSEA) showed that the transcriptional pattern induced by ↑[H^+^] exposure was similar to that of memory T cells (Fig. 1g and Extended Data Fig. 1f). Furthermore, quantitative polymerase chain reaction (qPCR) analysis confirmed increased mRNA expression of *BACH2*, *KLF2*, *LEF1* and *TCF7* following ↑[H^+^] treatment (Fig. 1h). Collectively, these observations support the fact that ↑[H^+^] treatment greatly shapes the transcriptional profiles of T cells to establish stemness. Of note, we found that ↑[H^+^] treatment inhibited T cell proliferation and skewed the cell-cycle distribution towards the G1 phase and away from the S phase (Supplementary Fig. 1a–c). We next examined T cell activation upon ↑[H^+^] treatment during TCR stimulation and found that ↑[H^+^] exposure has no significant effect on the expression of T cell activation markers or cell size (Supplementary Fig. 2a,b). Additionally, we further confirmed that ↑[H^+^] exposure after T cell activation could still induce a stem-like state of T cells (Supplementary Fig. 2c–f). These findings rule out the possibility that T cells exposed to ↑[H^+^] are simply refractory to stimulation, as opposed to adopting a stem-like state.

Previous reports have shown that the acidic milieu acutely inhibits the cytolytic activity of T cells both in vitro and in vivo^23,31^. We also found that short-term ↑[H^+^] exposure impaired the production of cytokines but had little impact on the stem-like phenotype in T cells (Extended Data Fig. 1g–i and Supplementary Fig. 3a–c). Furthermore, the inhibitory effects of cytokine production by ↑[H^+^] exposure are transient and reversible because the removal of ↑[H^+^] rapidly restores the cytokine production by T cells (Extended Data Fig. 1h). Thus, inhibition of T cell effector function by the acidic milieu is very rapid, whereas reprogramming of T cell stemness requires prolonged ↑[H^+^] exposure. Because lactate is present in solution either in its undissociated form (lactic acid) or as an ion salt (sodium lactate), we next sought to determine whether sodium lactate displayed similar effects on T cell stemness and found that sodium lactate also promoted stemness signatures, although its efficacy was much lower than that of lactic acid treatment (10 mM) (Extended Data Fig. 1j,k and Supplementary Fig. 4a–f). These findings suggest both the importance of ↑[H^+^], and its difference from lactate ions treatment, in the induction of the stem-like phenotype of CD8^+^ T cells.

### Elevated [H^+^] triggers metabolic reprogramming

**Fig. 2:**
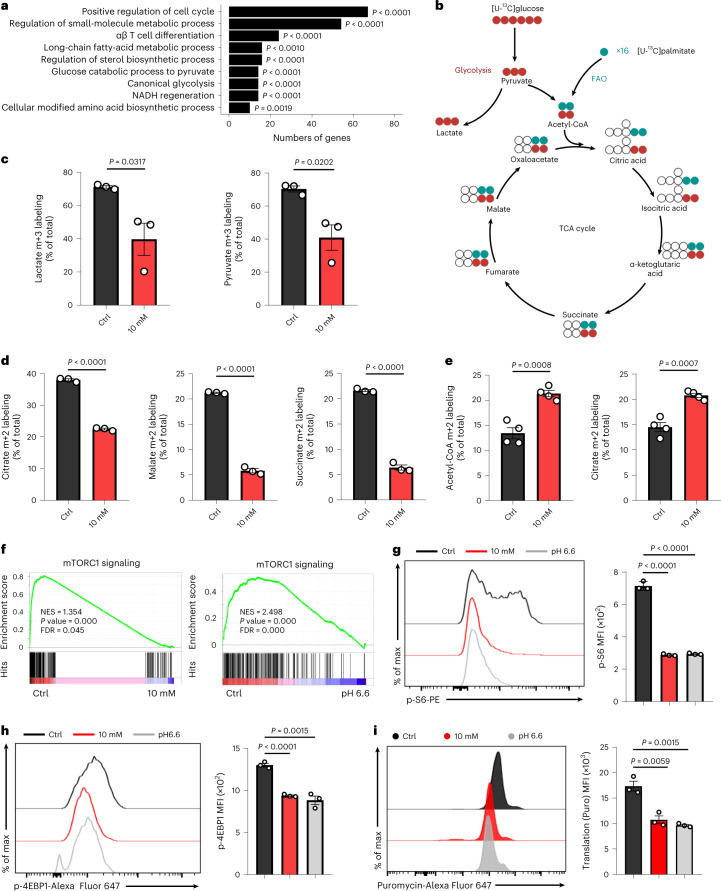
↑[H^+^] exposure triggers metabolic reprogramming and suppresses mTOR signaling. **a**, GO analysis using RNA-seq data, showing representative differentially expressed metabolic genes in control and lactic-acid-conditioned human T cells (adjusted *P* value < 4.23 × 10^–2^). **b**, Schematic of [^13^C_6_]glucose or [^13^C_16_]palmitate labeling patterns. **c**, Percentage of the indicated m+3 lactate out of total lactate or of m+3 pyruvate out of total pyruvate in T cells. *n* = 3 independent samples. **d**, Percentage of isotopomer for the TCA intermediates, such as citrate (m+2), malate (m+2) and succinate (m+2), derived from [^13^C_6_]glucose. *n* = 3 independent samples. **e**, Percentage of the indicated m+2 acetyl-CoA out of total acetyl-CoA or of m+2 citrate isotope out of total citrate in T cells from [^13^C_16_]palmitate. *n* = 4 independent samples. **f**, GSEA with statistical analysis of the gene set associated with mTORC1 signaling in control versus lactic-acid-conditioned (left) or pH 6.6-conditioned (right) human T cells. **g**,**h**, Flow cytometric analysis and quantification for S6 phosphorylated at Ser235 and Ser236 (**g**) and 4EBP1 phosphorylated at Thr37 and Thr46 (**h**) in human CD8^+^ T cells under the indicated conditions. *n* = 3 independent samples. **i**, Flow cytometric analysis and quantification of energy-intensive protein synthesis in controls or lactic-acid- or pH 6.6-conditioned human T cells. *n* = 3 independent samples. Data are presented as mean ± s.e.m. Statistical analyses were determined by one-sided Fisher exact test with Benjamini–Hochberg multiple-comparisons test (**a**) or unpaired two-tailed Student’s *t*-test (**c**–**e**,**g**–**i**). Nominal *P* values and FDRs were calculated with the default method in the GSEA software (**f**). Source data

**Extended Data Fig. 2:**
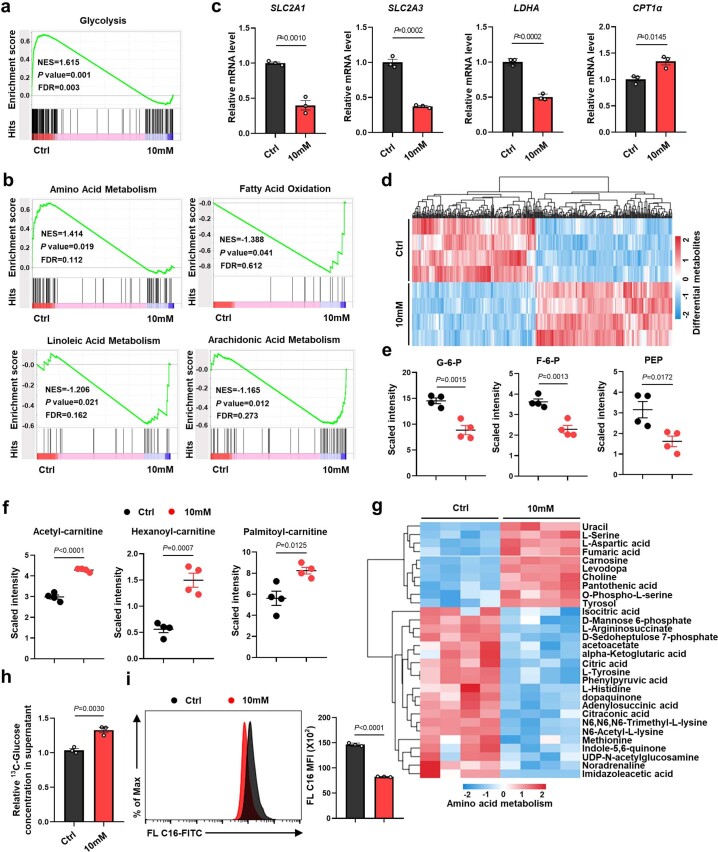
↑[H^+^] exposure limits nutrient uptake and results in T cell metabolic alterations. (**a**) GSEA plot of gene set associated with glycolysis in control versus lactic acid conditioned human T cells. (**b**) GSEA plots comparing enrichment of amino acid metabolism and fatty acid metabolism in control and lactic acid conditioned human T cells. (**c**) Quantitative mRNA expression of genes associated with glycolysis (*SLC2A1*, *SLC2A3*, *LDHA*) and fatty acid metabolism (*CPT1α*) in control and lactic acid conditioned human T cells. n = 3 independent samples. (**d**) Heat map showing the relative abundance of differential metabolites in human T cells under control and lactic acid conditions. (**e,f**) Quantification of glycolytic intermediates (**e**) and FAO metabolites (**f**) by liquid chromatography and mass spectrometry in human T cells under the indicated conditions (Data are from 4 replicates per condition). (**g**) Heat map depicting the abundance of metabolites of amino acid metabolism in control and lactic acid conditioned human T cells. (**h**) Relative glucose consumption by assessing the [^13^C_6_] glucose in the supernatant under control or lactic acid treatment. n = 3 independent samples. (**i**) Flow cytometric analysis and quantification for BODIPY FL C16 uptake in the indicated conditions. n = 3 independent samples. Data are presented as mean ± SEM. Statistical analyses were determined by unpaired two-tailed Student’s *t*-test (**c,e,f,h,i**). Nominal *P* value and FDR were calculated with default method of GSEA software (**a,b**). Source data

Beyond the distinct transcriptional programs, stem-like T cells also preferentially acquire unique metabolic attributes, including elevated FAO and restricted glycolytic metabolism^17,20,37^. To explore the metabolic features of long-term in vitro ↑[H^+^]-exposed T cells, we performed Gene Ontology (GO) enrichment analysis and found significant differences in the expression of genes related to metabolic pathways, such as small-molecule metabolism and glycolysis (Fig. 2a). Indeed, long-term ↑[H^+^]-conditioned T cells exhibited reduced glycolysis and amino acid metabolism in contrast to increased long-chain fatty acid metabolism (Extended Data Fig. 2a,b). Unlike long-term ↑[H^+^] exposure, short-term ↑[H^+^] treatment inhibited only glycolysis, but had no significant effects on FAO (Supplementary Fig. 5a). Quantitative PCR analysis further confirmed that the glycolysis genes *SLC2A1*, *SLC2A3* and *LDHA* were significantly decreased in ↑[H^+^]-exposed T cells (Extended Data Fig. 2c). Conversely, ↑[H^+^] conditioning promoted the expression of carnitine palmitoyltransferase 1α (encoded by *CPT1A*), a rate-limiting enzyme involved in FAO (Extended Data Fig. 2c). To further elucidate the metabolic alterations induced by ↑[H^+^], we performed an unbiased metabolomics analysis and found 285 distinct intermediates in T cells cultured with ↑[H^+^] versus controls (Extended Data Fig. 2d). In particular, exposure to ↑[H^+^] significantly decreased the glycolytic intermediates and certain essential amino acids instead of increasing multiple carnitine species, the activated form of fatty acids that is transferred to mitochondria for oxidation (Extended Data Fig. 2e–g). Metabolic flux analysis using [^13^C_6_]glucose or [^13^C_16_]palmitate demonstrated that ↑[H^+^] exposure drastically hindered [^13^C_6_]glucose incorporation into TCA intermediates and lactic acid while promoting [^13^C_16_]palmitate incorporation into acetyl-CoA and citrate, supporting the notion that extracellular acidosis suppresses glycolysis and enhances FAO in T cells (Fig. 2b–e). These ↑[H^+^]-conditioned T cells displayed limitations with respect to nutrient uptake, as evidenced by the decreased consumption of [^13^C_6_]glucose and absorption of lipid analogs (BODIPY FL C_16_), a finding that may be due to an increased electrochemical gradient (Extended Data Fig. 2h,i).

**Extended Data Fig. 3:**
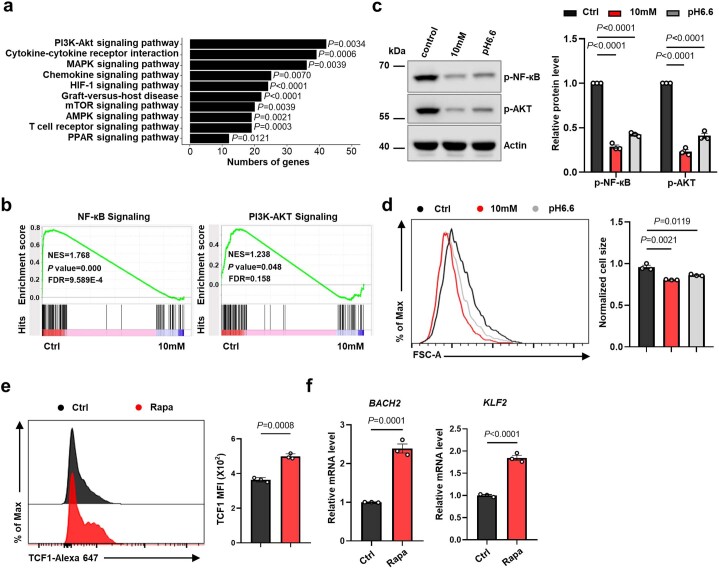
↑[H^+^] exposure suppresses mTOR signaling. (**a**) Gene ontology (GO) analysis from RNA-seq data showing several differentially expressed genes associated with signaling pathways in control versus lactic acid conditioned human T cells (*P* value adjusted < 4.27×10^−2^). (**b**) GSEA plots with statistical analysis of gene sets associated with NF-кB (left) and PI3K-AKT (right) signaling comparing control versus lactic acid conditioned human T cells. (**c**) Assessment and quantification of p-AKT (Ser^473^) and p-NF-кB (Ser^536^) by western blot in control, lactic acid and pH 6.6 conditioned human T cells. n = 3 independent samples. (**d**) Flow cytometric analysis and quantification of forward scatter in control, lactic acid and pH 6.6 conditioned human CD8^+^ T cells. n = 3 independent samples. (**e**) Representative histogram and quantification of TCF1 in Rapa-treated (10 nM) and control human T cells. n = 3 independent samples. (**f**) Quantitative mRNA expression of *BACH2* and *KLF2* in Rapa-treated human T cells. n = 3 independent samples. Data are presented as mean ± SEM. Statistical analyses were determined by one-sided Fisher exact test with Benjamini & Hochberg multiple-comparisons (**a**), two-way ANOVA with Tukey’s multiple-comparisons test (**c**) or unpaired two-tailed Student’s *t*-test (**d-f**). Nominal *P* value and FDR were calculated with default method of GSEA software (**b**). Source data

The limited nutrient uptake and cellular-metabolism switch could lead to the alterations of the signaling networks in T cells. GO enrichment analysis of T cells treated with lactic acid revealed that most of the affected genes are primarily involved with PI3K–AKT, mTOR and TCR signaling (Extended Data Fig. 3a). mTOR signaling plays a central role in integrating immune signals and metabolic cues for the proper activation of T cells, and inhibition of mTOR signaling skews cells towards formation of memory CD8^+^ T cells instead of effector differentiation^38–40^. Our GSEA analysis indicated that the activity of both AKT–mTOR and NF-кB signaling in ↑[H^+^]-conditioned T cells was reduced compared with that in control T cells (Fig. 2f and Extended Data Fig. 3b). This was further confirmed by the decreased phosphorylation of S6 (at Ser235 and Ser236), 4EBP1 (at Thr37 and Thr46), AKT (at Ser473) and NF-кB (at Ser536) in ↑[H^+^]-conditioned T cells (Fig. 2g,h and Extended Data Fig. 3c). mTOR is known to be a critical regulator of diverse biological processes, including cell size, energy generation and protein synthesis^41^. Indeed, we found that the cell size of ↑[H^+^]-conditioned T cells was reduced as compared with that of control T cells (Extended Data Fig. 3d). We next assessed bioenergy-dependent protein synthesis in ↑[H^+^]-conditioned T cells using SCENITH, a newly developed method that uses puromycin incorporation as a readout of protein-synthesis levels^42^. As expected, ↑[H^+^] exposure suppressed energy-intensive protein synthesis (Fig. 2i), suggesting that energy metabolism in CD8^+^ T cells was altered. These results are consistent with previous findings that inhibition of mTOR activity by rapamycin increased the expression of stemness-associated transcriptional factors (Extended Data Fig. 3e,f). Taking our results together, we conclude that long-term ↑[H^+^] exposure orchestrates a metabolic switch and suppresses mTOR activity, thereby facilitating the acquisition and maintenance of T cell stemness.

### ↑[H^+^]-mediated restriction of methionine metabolism preserves epigenetic stemness

**Fig. 3:**
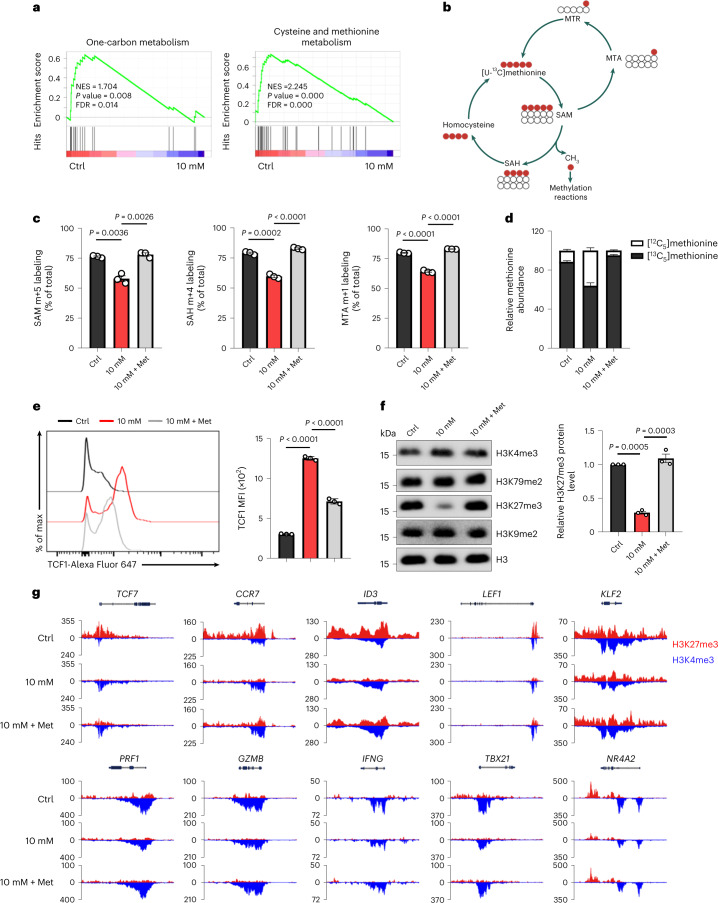
Increased [H^+^] alters T cell methionine metabolism to preserve epigenetic stemness. **a**, GSEA plot of the gene set associated with one-carbon metabolism and cysteine and methionine metabolism in control versus lactic-acid-conditioned human T cells. **b**, Schematic of [^13^C_5_]methionine labeling patterns. **c**, Percentage of intracellular SAM (m+5), SAH (m+4) and MTA (m+1) derived from [^13^C_5_]methionine, out of their respective total pools, in T cells cultured in control conditions or with 10 mM lactic acid or 10 mM lactic acid supplemented with methionine (10 mM + Met). *n* = 3 independent samples. **d**, Relative abundance of [^12^C_5_]methionine and [^13^C_5_]methionine in T cells. **e**, Representative histogram and quantification of TCF1 in human CD8^+^ T cells cultured in various conditions. *n* = 3 independent samples. **f**, Effects of methionine supplementation on histone methylation in human T cells. H3K4me3, histone H3 trimethylated at K4; H3K79me2, H3 dimethylated at K79; H3K27me3, H3 trimethylated at K27; H3K9me2, H3 dimethylated at K9. *n* = 3 independent samples. **g**, Genome track view of representative gene loci showing H3K27me3 (red, above the line) or H3K4me3 (blue, below the line) peaks. CUT&Tag-seq data are from two independent samples. Data are presented as mean ± s.e.m. Nominal *P* values and FDRs were calculated with the default method of the GSEA software (**a**). Statistical analyses were done using two-way analysis of variance (ANOVA) with Tukey’s multiple-comparisons test (**c**–**f**). Source data

**Extended Data Fig. 4:**
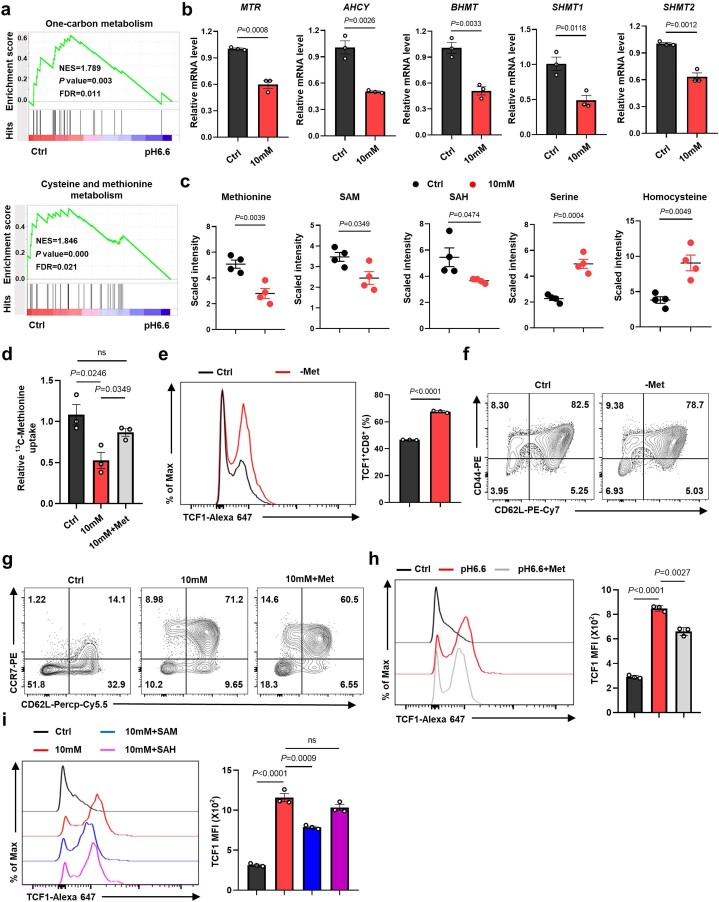
Exogenous methionine supplementation reprograms ↑[H^+^]-induced restriction of methionine metabolism and stemness-like phenotype. (**a**) GSEA plot of genes set associated with one-carbon metabolism and cysteine and methionine metabolism in control versus pH 6.6 conditioned human T cells. (**b**) Quantitative mRNA expression of genes associated with one-carbon metabolism (*MTR*, *AHCY*, *BHMT*, *SHMT1*, *SHMT2*) in control and 10 mM lactic acid conditioned human T cells. n = 3 independent samples. (**c**) Quantification of methionine metabolic intermediates (Methionine, SAM, SAH, Serine, Homocysteine) by liquid chromatography and mass spectrometry under the indicated conditions (Data are from 4 replicates per condition). (**d**) Relative methionine uptake by assessing the [^13^C_5_] methionine in the supernatant in different conditions. n = 3 independent samples. (**e**) Left: Representative histogram of TCF1 in Ctrl (100 μM methionine) and -Met (20 μM methionine) conditioned human CD8^+^ T cells. Right: The statistical percentage of TCF1^+^CD8^+^ T cells population. n = 3 independent samples. (**f**) Flow cytometric analysis of CD44^+^CD62L^+^ population in Ctrl (100 μM methionine) and -Met (20 μM methionine) conditioned T cells. (**g**) Representative FACS plots show percentage of CCR7^+^CD62L^+^ population in human CD8^+^ T cells cultured with the indicated conditions. (**h**) Representative histogram and quantification of TCF1 in human CD8^+^ T cells cultured with control, pH 6.6 or pH 6.6 supplemented with methionine conditions. n = 3 independent samples. (**i**) Representative histogram and quantification of TCF1 in human CD8^+^ T cells cultured with control, 10 mM lactic acid, or 10 mM lactic acid supplemented with SAM or SAH conditions. n = 3 independent samples. Data are presented as mean ± SEM. Nominal *P* value and FDR were calculated with default method of GSEA software (**a**). Statistical analyses were determined by unpaired two-tailed Student’s *t*-test (**b,c,e**) or two-way ANOVA with Tukey’s multiple-comparisons test (**d**,**h,i**). ns, non-significant. Source data

Accumulating lines of evidence suggest that one-carbon metabolism dictates T cell fate decisions and shapes their functional states^43,44^. Our RNA-seq analysis showed poor enrichment for the one-carbon metabolic process and methionine cycle signature in T cells exposed to long-term, rather than short-term,↑[H^+^] treatment (Fig. 3a, Extended Data Fig. 4a, and Supplementary Fig. 5b). Accordingly, ↑[H^+^] exposure inhibited the expression of genes encoding enzymes related to methionine cycle (*MTR*, *AHCY* and *BHMT*) as well as folate metabolism (*SHMT1* and *SHMT*2) (Extended Data Fig. 4b). Further metabolomics analysis revealed that T cells cultured in lactic acid showed a marked decrease of intracellular metabolites involved in the methionine cycle, including methionine, *S*-adenosylmethionine (SAM) and *S*-adenosylhomocysteine (SAH), but increased levels of serine and homocysteine (Extended Data Fig. 4c). [^13^C_5_]methionine tracing further confirmed that the uptake and intracellular abundance of methionine, ^13^C-labeled intracellular SAM (m+5), SAH (m+4) and 5′-methyl-thioadenosine (MTA, m+1) were significantly reduced in ↑[H^+^]-exposed T cells (Fig. 3b–d and Extended Data Fig. 4d). Of note, exogenous methionine supplementation restored the uptake and intracellular abundance of [^13^C_5_]methionine as well as the relevant intermediates in ↑[H^+^]-exposed T cells (Fig. 3c,d and Extended Data Fig. 4d), suggesting that exogenous methionine supplementation could restore methionine uptake and metabolism of ↑[H^+^]-exposed T cells. We next investigated whether methionine restriction can drive T cell stemness. We found that methionine deprivation indeed promoted the induction of the TCF1^+^CD8^+^ T cell population but had no effect on the expression of CD62L and CD44 (Extended Data Fig. 4e,f). To further study the role of methionine metabolism in ↑[H^+^]-induced T cell stemness, we cultured T cells with ↑[H^+^] medium supplemented with methionine, SAM, or SAH. The phenotype of T cell stemness induced by extracellular acidosis was indeed partially prohibited by supplementation with methionine or SAM, but not SAH (Fig. 3e and Extended Data Fig. 4g–i).

**Extended Data Fig. 5:**
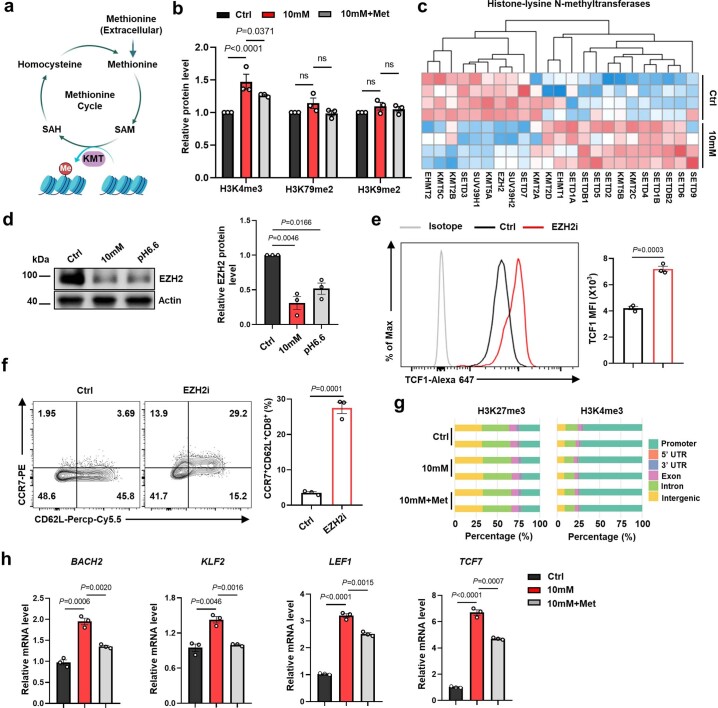
↑[H^+^] exposure triggers the changes of epigenetic patterns in T cells. (**a**) Schematic of the methionine cycle. (**b**) Effects of methionine supplementation on histone methylation in human T cells. n = 3 independent samples. (**c**) Heat map of genes associated with histone methyltransferases in control or lactic acid conditioned human T cells. (**d**) EZH2 expression in ↑[H^+^]-exposed T cells. n = 3 independent samples. (**e,f**) T cells were activated for 3 days and then treated with EZH2 inhibitor (GSK126, 10 μM) for 9 days. Effects of GSK126 treatment on the TCF1 expression (**e**) and generation of CCR7^+^CD62L^+^ T cell population (**f**). n = 3 independent samples. (**g**) Bar plot showing the percentage of H3K4me3 or H3K27me3 peaks at promoter regions (±1 kb from the TSS), gene body regions, or intergenic regions. UTR, untranslated region; TSS, transcription start site. (**h**) Quantitative mRNA expression of transcription factors associated with T cell stemness (*BACH2*, *KLF2*, *LEF1*, *TCF7*) under the indicated conditions. n = 3 independent samples. Data are presented as mean ± SEM. Statistical analyses were determined by two-way ANOVA with Tukey’s multiple-comparisons test (**b**,**h**) or unpaired two-tailed Student’s *t*-test (**d-f**). ns, non-significant. Source data

Intracellular methionine is converted into SAM, an important methyl-group donor for DNA and histone methylation reactions (Extended Data Fig. 5a). Thus, we characterized histone methylation patterns and found that elevated [H^+^] greatly reduced total H3K27me3 levels in T cells, but did not have a significant effect on the expression of other histone methylation markers (Fig. 3f and Extended Data Fig. 5b). As expected, methionine supplementation to T cells under ↑[H^+^] exposure restored the total level of H3K27me3 expression (Fig. 3f). The specific reduction of H3K27me3 level observed in T cells that underwent long-term↑[H^+^] treatment prompted us to hypothesize that ↑[H^+^] exposure selectively regulates the methyltransferase specific for the deposition of H3K27me3. Indeed, we observed significantly reduced expression of EZH2, a key methyltransferase for H3K27me3, both at the mRNA and protein level in ↑[H^+^]-exposed T cells (Extended Data Fig. 5c,d). Furthermore, inhibition of EZH2 activity by a highly specific inhibitor, GSK126, increased the expression of TCF1, as well as the percentage of CCR7^+^CD62L^+^CD8^+^ T cells (Extended Data Fig. 5e,f), which was in line with a previous report that EZH2 could regulate memory-T cell potential through selective epigenetic modifications^45^. To identify genome-wide changes in epigenetic patterns of ↑[H^+^]-induced T cell stemness, we performed the CUT&Tag-seq assay and found minimal changes in the proportionality of H3K4me3 and H3K27me3 deposition among different treated groups along the promoter, gene body and intergenic regions (Extended Data Fig. 5g). Specifically, epigenomic profiling revealed a decreased level of repressive histone marker H3K27me3 at memory-associated gene loci (for example, *TCF7*, *CCR7*, *ID3*, *LEF1* and *KLF2*) in long-term ↑[H^+^]-treated T cells (Fig. 3g). Importantly, the addition of methionine under ↑[H^+^] exposure partially restored the H3K27me3 level at the above loci, as well as their gene expression, suggesting an important role for methionine in the epigenetic modulation of these memory-associated genes (Fig. 3g and Extended Data Fig. 5h). Consistent with a previous study^14^, effector genes such as *PRF1*, *GZMB*, *IFNG*, *TBX21* and *NR4A2* favorably acquired an activating histone-methylation pattern (H3K4me3^hi^ and H3K27me3^lo^) (Fig. 3g). Of note, the occupancies of H3K4me3 at promoter regions of these effector genes were impaired in T cells cultured under ↑[H^+^] exposure and were restored by methionine supplementation (Fig. 3g). Overall, these findings indicate that ↑[H^+^]-mediated disruption of the methionine cycle promotes epigenetic preservation of T cell stemness.

**Extended Data Fig. 6:**
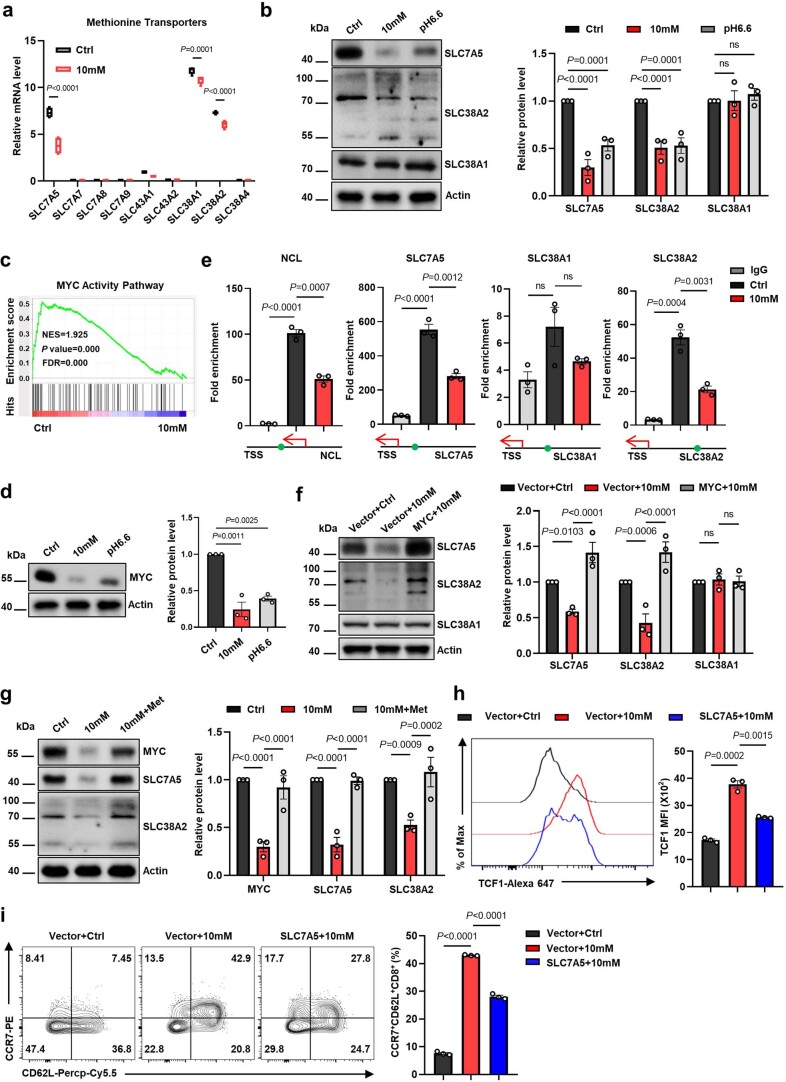
MYC-SLC7A5 axis is involved in the direct regulation of ↑[H^+^]-induced methionine restriction and stemness-like status. (**a**) Quantitative mRNA expression of methionine transporters (*SLCs*) in control and lactic acid conditioned human T cells. Box plots include minima, maxima and the centre line (median); box limits show the upper and lower quartiles; whiskers are 1.5× the interquartile range. Data are from 4 replicates per condition. (**b**) Assessment and quantification of the expression of methionine transporters in ↑[H^+^]-exposed T cells. n = 3 independent samples. (**c**) GSEA plot of genes set associated with MYC activity pathways in control versus lactic acid conditioned human T cells. (**d**) Assessment and quantification of the expression of MYC in ↑[H^+^]-exposed T cells. n = 3 independent samples. (**e**) Binding of MYC to the promoter of *SLC7A5*, *SLC38A2*, *SLC38A1* and *NCL* (a well-known MYC target used as a positive control), analyzed by ChIP assays in T cells cultured with control or lactic acid conditions. n = 3 independent samples. (**f**) Effects of overexpression of MYC on methionine transporters under ↑[H^+^] exposure. n = 3 independent samples. (**g**) Methionine supplementation in ↑[H^+^] conditions for 12 days to assess the expression of MYC and methionine transporters in T cells. n = 3 independent samples. (**h,i**) Transfected T cells were cultured in different conditions for 9 days. Effects of overexpression of SLC7A5 on the generation of TCF1 expression (**h**) and CCR7^+^CD62L^+^ population (**i**). n = 3 independent samples. Data are presented as mean ± SEM. Statistical analyses were determined by two-way ANOVA with Sidak’s multiple-comparisons test (**a**), two-way ANOVA with Tukey’s multiple-comparisons test (**b**,**f,g**–**i**) or unpaired two-tailed Student’s *t*-test (**d,e**). Nominal *P* value and FDR were calculated with default method of GSEA software (**c**). ns, non-significant. Source data

**Extended Data Fig. 7:**
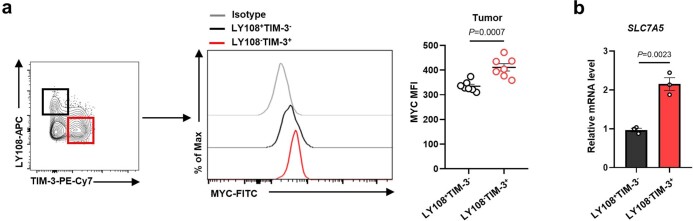
The expression of MYC and SLC7A5 in different subpopulations of CD8^+^ T cells within tumors. (**a**) MYC expression in tumoral LY108^+^TIM-3^-^ and LY108^−^TIM-3^+^ CD8^+^ T cell populations from B16-OVA tumor-bearing C57BL/6N mice (n = 7 mice). (**b**) The mRNA level of *SLC7A5* in LY108^+^TIM-3^-^ and LY108^−^TIM-3^+^ populations from B16-OVA tumor (n = 7 mice). Data are presented as mean ± SEM. Statistical analyses were determined by unpaired two-tailed Student’s *t*-test. Source data

Several methionine transporters (SLCs), including SLC7A5, SLC38A1, SLC38A2 and SLC43A2, can mediate methionine transportation^46^. To investigate how ↑[H^+^] exposure decreases methionine uptake and metabolism in T cells, we analyzed the expression patterns of distinct SLCs and found that ↑[H^+^] dramatically downregulated the expression of SLC7A5 and SLC38A2, but had little influence on SLC38A1 expression (Extended Data Fig. 6a,b). In addition, ↑[H^+^] exposure reduced the expression and activity of MYC (Extended Data Fig. 6c,d), which has been reported to regulate the expression of methionine transporters like SLC7A5 (ref. ^47^). We further demonstrated a high occupancy of MYC on the *SLC7A5* promoter and to a lesser extent on the *SLC38A2* promoter (Extended Data Fig. 6e). Importantly, significantly reduced MYC binding to these loci was observed in long-term ↑[H^+^]-treated T cells (Extended Data Fig. 6e). Consistent with these observations, overexpression of MYC remarkably restored the expression of SLC7A5 and SLC38A2 in ↑[H^+^]-exposed T cells (Extended Data Fig. 6f). These results support the important role of MYC in reduced expression of methionine transporters upon ↑[H^+^] exposure. Interestingly, we found that the supplementation of methionine can also restore the expression of MYC and SLC7A5 in ↑[H^+^]-exposed T cells (Extended Data Fig. 6g), suggesting that methionine supplementation might restore the methionine-uptake ability of T cells by upregulating methionine transporter SLC7A5 expression in a MYC-dependent manner. A previous study has shown that SLC7A5 is the most abundant methionine transporter in activated T cells^47^. This, together with our previous findings, suggests that SLC7A5 might play an important role in ↑[H^+^]-induced methionine restriction and the maintenance of a stem-like state. Indeed, we found that overexpression of SLC7A5 partially impaired the ↑[H^+^]-induced stem-like phenotype (Extended Data Fig. 6h,i), further supporting the involvement of the methionine transporter SLC7A5 in ↑[H^+^]-induced T cell memory-like phenotype. By analyzing different tumor-infiltrating CD8^+^ T cell subpopulations, we found reduced expression of both MYC and SLC7A5 in memory-like CD8^+^ TILs (Extended Data Fig. 7a,b), which is in line with our in vitro findings. Thus, these findings suggest that the MYC–SLC7A5–methionine axis could potentially contribute to preserving the memory-like status of TILs.

### Exposure to ↑[H^+^] maintains mitochondrial fitness

**Fig. 4:**
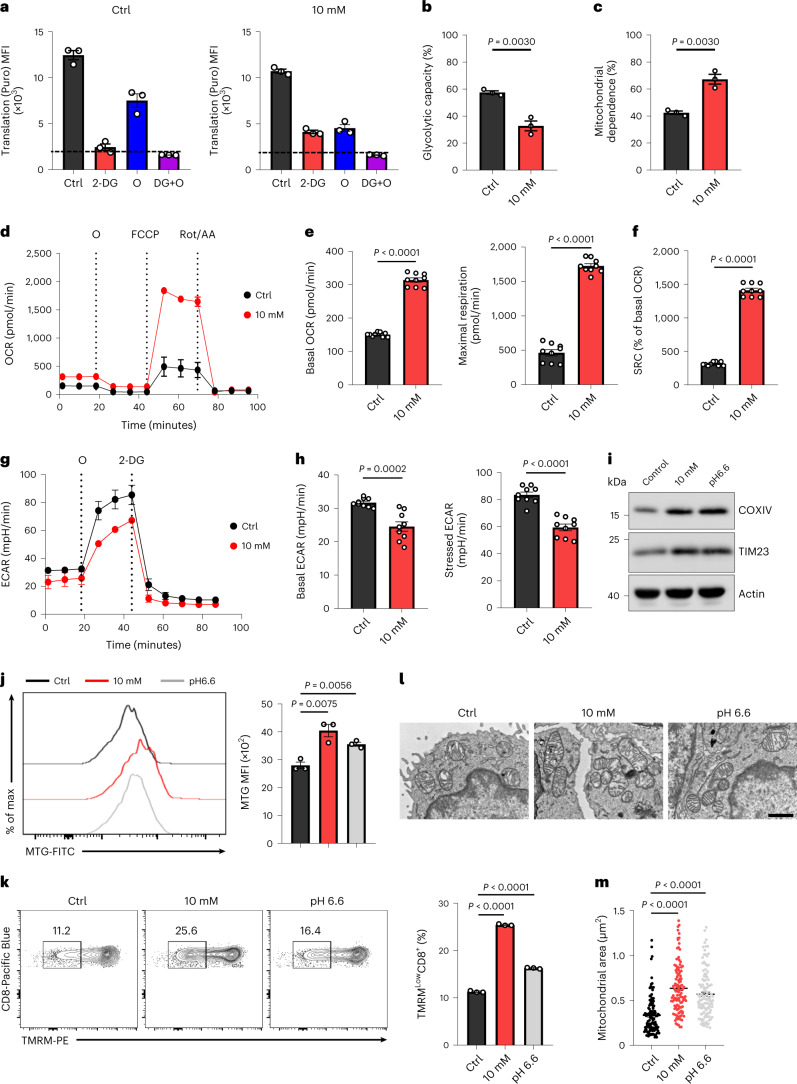
Mitochondrial fitness is sustained in T cells exposed to ↑[H^+^]. **a**, SCENITH analysis of the human T cells in control or lactic-acid-conditioned T cells. Representative translation level (anti-Puro) is shown (*n* = 3 independent samples). The dashed line represents the background level obtained after 2-deoxy-d-glucose + oligomycin (2-DG+O) treatment. **b**,**c**, Quantitative analysis of glycolytic capacity (**b**) and mitochondrial dependence (**c**) within **a**. *n* = 3 independent samples. **d**–**f**, OCR (**d**) of control and lactic-acid-conditioned T cells was measured in real-time under basal conditions in response to the indicated inhibitors. FCCP, carbonyl cyanide-*p*-trifluoromethoxyphenylhydrazone; ROT/AA, rotenone and antimycin A. Representative statistical analysis of basal OCR (**e**), maximal respiration (**e**) and SRC (**f**). *n* = 9 tests; 3 independent samples were analyzed and each sample was measured 3 times. **g**,**h**, ECAR (**g**) of control or lactic-acid-conditioned T cells measured in response to the indicated inhibitors. Representative statistical analysis of basal ECAR and stressed ECAR (**h**). *n* = 9 tests; 3 independent samples were detected and each sample was measured 3 times. **i**, Immunoblot analysis of COXIV and TIM23 in human T cells under the indicated conditions. Actin was used as a loading control. **j**,**k**, Representative histograms or contour plots and statistical analysis of mitochondrial mass (MTG) (**j**) and mitochondrial membrane potential (TMRM) (**k**), respectively, in the control or lactic-acid- or pH 6.6-conditioned human T cells. *n* = 3 independent samples. **l**,**m**, Representative mitochondrial morphology of T cells cultured in the control condition, lactic acid or the pH 6.6 condition for 12 days, analyzed by EM (scale bar, 1 μM) (**l**). The area of individual mitochondria in T cells (**m**), *n* = 45 cells. Data are presented as mean ± s.e.m. Statistical analyses were done using unpaired two-tailed Student’s *t*-test. Source data

**Extended Data Fig. 8:**
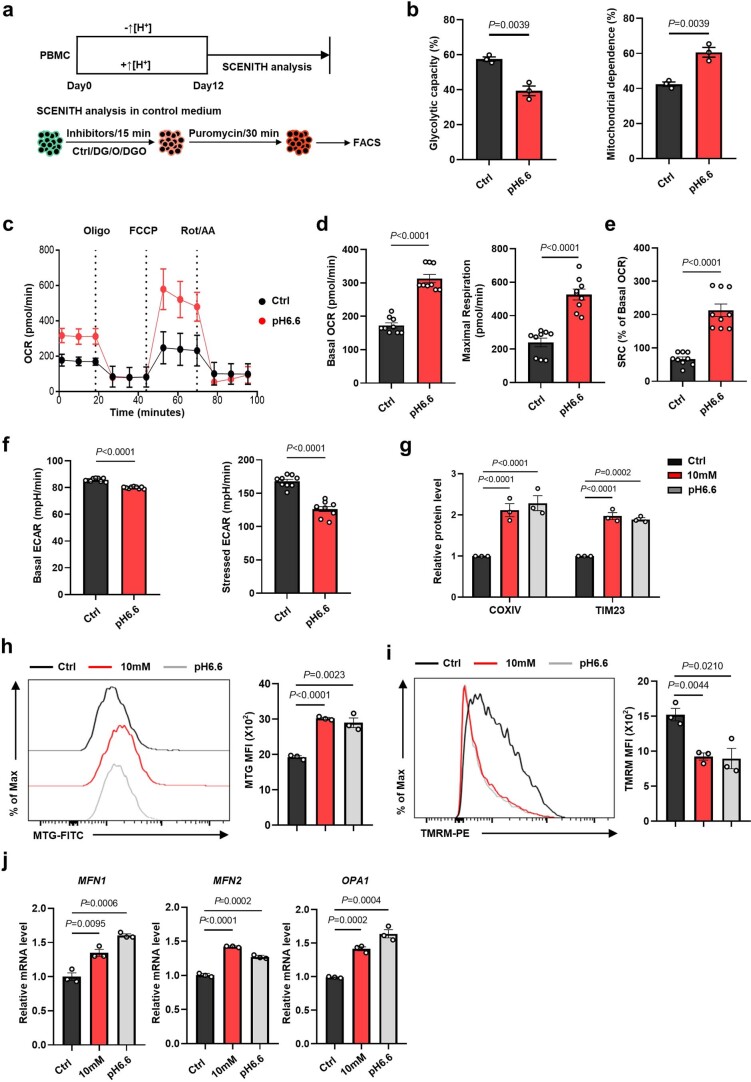
Increased [H^+^] enhances mitochondrial activity. (**a**) Outline of the SCENITH method. (**b**) Representative statistical analysis of glycolytic capacity and mitochondrial dependence in control and pH 6.6 conditioned human T cells. n = 3 independent samples. (**c-e**) OCR (**c**) of control and pH 6.6 conditioned T cells were measured in real time under basal conditions in response to the indicated inhibitors. Representative statistical analysis of basal OCR (**d**), maximal respiration (**d**) and SRC (**e**). n = 9, 3 independent samples were detected and each sample was measured three times. (**f**) Representative statistical analysis of basal ECAR and stressed ECAR in control and pH 6.6 conditioned T cells. n = 9, 3 independent samples were detected and each sample was measured three times. (**g**) Statistics of COXIV and TIM23 in human T cells under indicated conditions. n = 3 independent samples. (**h,i**) Representative histograms and statistical analysis of mitochondrial mass (**h**) and mitochondrial membrane potential (**i**) in the control, lactic acid or pH 6.6 conditioned mouse OT-I T cells. n = 3 independent samples. (**j**) Quantitative mRNA expression of *MFN1*, *MFN2*, *OPA1* in T cells. n = 3 independent samples. Data are presented as mean ± SEM. Statistical analyses were determined by unpaired two-tailed Student’s *t*-test (**b**,**d**–**f,h**–**j**) or two-way ANOVA with Tukey’s multiple-comparisons test (**g**). Source data

Given the reduced energy-intensive protein synthesis in ↑[H^+^]-conditioned T cells, we hypothesized that long-term exposure to ↑[H^+^] might result in considerable changes in cellular energetic metabolism. Both control and ↑[H^+^]-conditioned T cells were analyzed using SCENITH^42^ in order to calculate glucose dependence, mitochondrial dependence, glycolytic capacity and FAO and amino acid oxidation capacity (Fig. 4a and Extended Data Fig. 8a). In accordance with our metabolomics and isotope-tracing results, we observed increased mitochondrial metabolism in ↑[H^+^]-exposed T cells, whereas glycolysis rates were reduced (Fig. 4b,c and Extended Data Fig. 8b), indicating a reprograming of intracellular energetic metabolism in ↑[H^+^]-conditioned T cells. Seahorse analysis further revealed that ↑[H^+^]-conditioned T cells showed a higher oxygen consumption rate (OCR) as well as SRC, a cardinal feature of long-lived memory CD8^+^ T cells^22^ and a lower extracellular acidification rate (ECAR) (Fig. 4d–h and Extended Data Fig. 8c–f). As increased mitochondrial mass/fusion and decreased mitochondrial membrane potential (Δψm) are important for the maintenance of T cell stemness^48–50^, we further examined the quantity and quality of mitochondria of ↑[H^+^]-conditioned T cells and found that ↑[H^+^] treatment enhanced mitochondrial mass in both human and mouse T cells (Fig. 4i,j and Extended Data Fig. 8g,h), which also maintain a lower Δψm (Fig. 4k and Extended Data Fig. 8i). Ultrastructure analysis by electron microscopy (EM) revealed that ↑[H^+^]-exposed T cells had large, densely packed mitochondria dispersed in the cytoplasm and had many tight, narrow cristae as compared with control T cells, which is in line with published results related to mitochondria morphology in memory T cells (Fig. 4l,m). Consistent with this finding, we also observed increased gene expression of several critical regulators of mitochondrial fusion in ↑[H^+^]-conditioned T cells (Extended Data Fig. 8j), which has been suggested to play a necessary role for memory T cell generation^50^. Altogether, these findings indicate that ↑[H^+^] exposure enhances mitochondrial mass/fusion and fitness to promote the formation of stem-like T cells.

### ↑[H^+^] exposure enhances CD8^+^ T cell anti-tumor activity

**Fig. 5:**
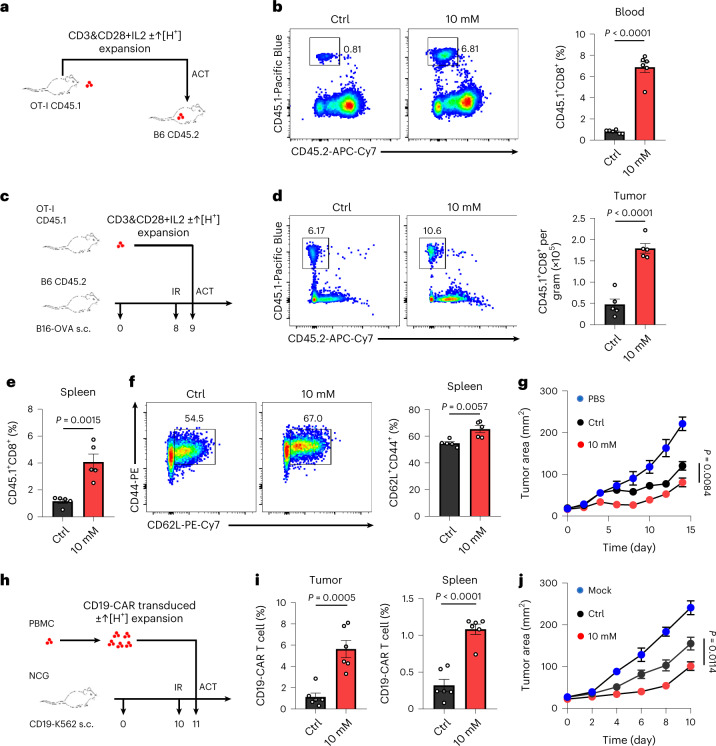
↑[H^+^]-expanded T cells display enhanced anti-tumor activity. **a**,**b**, Control or lactic-acid-expanded CD8^+^ T cells were analyzed for persistence after adoptive transfer (*n* = 6 mice). Freshly isolated mouse CD45.1^+^ OT-I T cells were activated with mouse IL-2 and plate-bound anti-mouse CD3 and anti-mouse CD28 antibodies for 2 days and then maintained in a culture medium with mouse IL-2 until adoptive transfer (CD3&CD28+IL-2). A schematic of the animal experiment (**a**) as well as representative FACS plots and statistical analysis of CD45.1^+^ and CD45.2^+^ T cells in the blood are shown in (**b**). **c**–**g**, CD45.1^+^ OT-I T cells were expanded in control or lactic acid medium for 7 days and transferred into B16-OVA-tumor-bearing mice, and the infiltration of ratio was evaluated (*n* = 5 mice). A schematic of animal experiment using B16-OVA tumor-bearing mice (**c**), as well as representative FACS plots (left) and statistics for the number (right) of transferred CD45.1^+^ OT-I T cells in the tumor (**d**). s.c., subcutaneous injection. Statistics for the percentage of CD45.1^+^ T cells (**e**) and representative data (left) and statistics for the percentage (right) of CD62L^+^CD44^+^CD45.1^+^ T cells (**f**) in the spleen are shown. Tumor growth curve (**g**) (*n* = 5 mice, day 14). **h**–**j**, CD19-CAR T cells were expanded in control or lactic acid medium for 12 days and transferred into CD19-overexpressing K562 tumor-bearing NCG mice, and the infiltration ratio in tumor and spleen were evaluated (*n* = 6 mice). A schematic of the animal experiment (**h**), representative percentage of transferred T cells in the tumor and spleen (**i**) and tumor growth curves (**j**) are shown (*n* = 6 mice, day 10). Data are presented as mean ± s.e.m. Statistical analyses were done using unpaired two-tailed Student’s *t*-test. Source data

**Extended Data Fig. 9:**
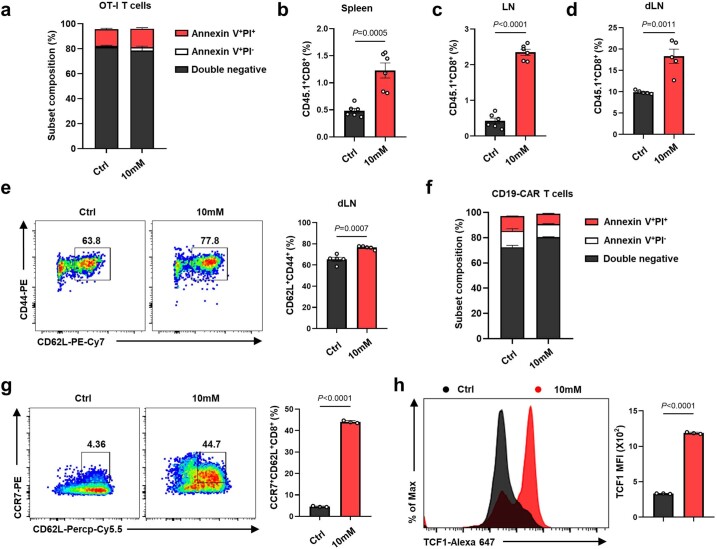
Exposure to ↑[H^+^] enhances T cell persistence. (**a**) Representative data and statistical analysis used to define the percentages of live (Annexin V^−^PI^−^), early apoptotic (Annexin V^+^PI^−^) and late apoptotic and necrotic populations (Annexin V^+^PI^+^) of OT-I T cells expanded in indicated conditions at day 7. (**b,c**) Splenocytes and lymphocytes from mice that received control or lactic acid expanded CD8^+^ T cells were analyzed for persistence after adoptive transfer (n = 6 mice). Representative percentage and statistics analysis of CD45.1^+^ T cell in the spleen (**b**) and lymph nodes (**c**) are shown. (**d,e**) Draining lymph nodes from B16-OVA tumor-bearing mice that received control or lactic acid expanded CD8^+^ T cells were analyzed for persistence after adoptive transfer. (n = 5 mice). Representative percentage and statistics analysis of CD45.1^+^ (**d**) and CD62L^+^CD44^+^ (**e**) T cells in the lymph nodes are shown. (**f**) Representative data and statistical analysis used to define the percentages of live (Annexin V^−^PI^−^), early apoptotic (Annexin V^+^PI^−^) and late apoptotic and necrotic populations (Annexin V^+^PI^+^) of CD19-CAR transduced human T cell expanded in indicated conditions for 12 days. (**g,h**) Representative FACS plots of CD62L^+^CCR7^+^ (**g**) and histograms and statistical analysis of TCF1 (**h**) in CD19-CAR transduced human T cell expanded in indicated conditions for 12 days. n = 3 independent samples. Data are presented as mean ± SEM. Statistical analyses were determined by unpaired two-tailed Student’s *t*-test. Source data

Stem-like T cells favor engraftment, expansion and anti-tumor efficacy in adoptive immunotherapy^51^. To investigate whether long-term in vitro ↑[H^+^]-maintained CD8^+^ T cells exhibit enhanced expansion or persistence upon transfer, CD45.1^+^ OT-I T cells that were expanded in control or ↑[H^+^]-conditioned medium were adoptively transferred into CD45.2^+^ C57BL/6N mice with no implanted tumor (Fig. 5a). Although slightly increased numbers of apoptotic cells were detected upon ↑[H^+^] conditioning (Extended Data Fig. 9a), there was a significantly higher percentage of T cells in the peripheral blood of mice transferred with T cells expanded in the ↑[H^+^] condition as compared with those expanded in control medium (control T cells) (Fig. 5b). Similarly, significantly higher frequencies of T cells in the spleen and lymph nodes (LNs) were detected (Extended Data Fig. 9b,c). In addition, we found significantly increased T cell accumulation in tumors, spleen and draining lymph nodes in B16-OVA tumor-bearing mice in which ↑[H^+^]-expanded cells had been adoptively transfererd as compared with that in mice that received control cells, suggesting that CD8^+^ T cells maintained in ↑[H^+^] have improved persistence (Fig. 5c–e and Extended Data Fig. 9d). In line with these findings, the percentage of memory T cells was significantly increased in the spleens and LNs of mice that received ↑[H^+^]-conditioned T cells (Fig. 5f and Extended Data Fig. 9e). Notably, ↑[H^+^]-expanded OT-I T cells exhibited significantly delayed tumor growth compared with control T cells (Fig. 5g). We next investigated the therapeutic efficacy of ↑[H^+^]-expanded CD19 chimeric-antigen-receptor-modified (CAR) T cells with an in vivo tumor model in which CD19-K562 tumor cells were subcutaneously injected into the flanks of NCG mice (Fig. 5h). We found that ↑[H^+^] exposure also promoted CAR-T cell stemness but had no effect on apoptosis (Extended Data Fig. 9f), as evidenced by the significantly increased percentages of CCR7^+^CD62L^+^ stem-like CAR-T cells as well as the upregulated expression of TCF1 (Extended Data Fig. 9g,h). In addition, there was a higher rate of CAR-T cell accumulation in tumor sites and spleens of NCG mice after infusion with ↑[H^+^]-conditioned T cells (Fig. 5i). As expected, NCG mice that were adoptively transferred with CAR-T cells that had been expanded in ↑[H^+^] showed a greater clearance of implanted CD19^+^ tumor as compared with those that received CAR-T cells that had been expanded in control medium (Fig. 5j). Taken together, these data demonstrated that ↑[H^+^] treatment promotes in vivo persistence and anti-tumor activity of T cells.

### Exposure to ↑[H^+^] restricts T cell exhaustion

**Fig. 6:**
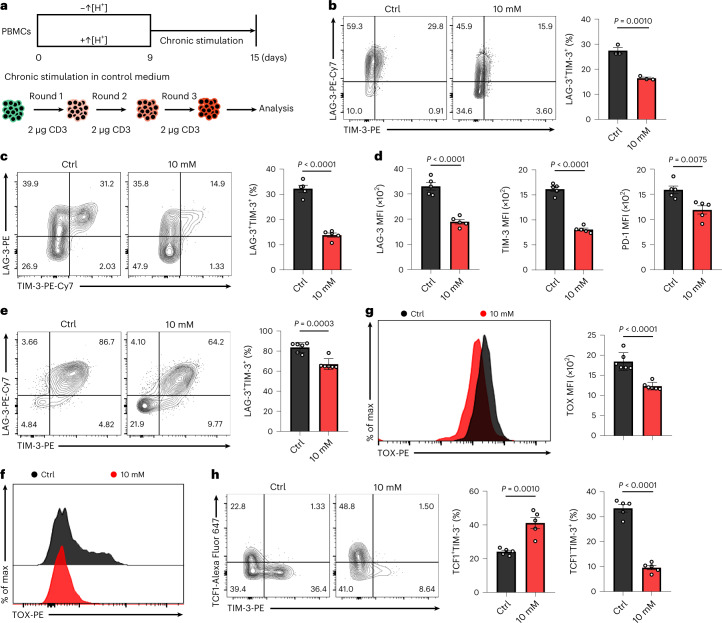
↑[H^+^] exposure restricts T cell exhaustion. **a**, Schematic of chronic stimulation of human T cells in vitro. **b**, Representative FACS plots for LAG-3 and TIM-3 in chronic stimulated human T cells cultured in control or lactic acid conditions. *n* = 3 independent samples. **c**, The expression of LAG-3 and TIM-3 in CD45.1^+^ TILs from B16-OVA tumor-bearing C57BL/6N mice (*n* = 5 mice). **d**, Quantification of the expression of LAG-3, TIM-3 and PD-1 in CD45.1^+^ TILs from B16-OVA-tumor-bearing C57BL/6N mice (*n* = 5 mice). **e**, The expression of LAG-3 and TIM-3 in tumor-infiltrating CD19-CAR T cells from CD19-K562 tumor-bearing NCG mice, as determined by flow cytometry (*n* = 6 mice). **f**, The expression of TOX in CD45.1^+^ TILs from B16-OVA tumor-bearing C57BL/6N mice (*n* = 5 mice). **g**, The histograms and statistical analysis of TOX in tumor-infiltrating CD19-CAR T cells from CD19-K562 tumor-bearing NCG mice (*n* = 6 mice). **h**, Left, representative flow cytometry plots for TIM-3 and TCF1 in CD45.1^+^ TILs from B16-OVA tumor-bearing C57BL/6N mice (*n* = 5 mice). Right, the percentage of TCF1^+^TIM-3^–^ or TCF1^–^TIM-3^+^ populations. Data are presented as mean ± s.e.m. Statistical analyses were done using unpaired two-tailed Student’s *t*-test. Source data

**Extended Data Fig. 10:**
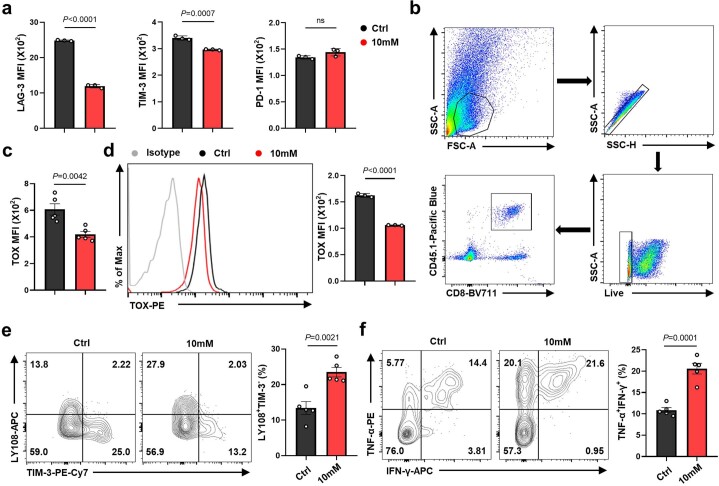
↑[H^+^]-expanded T cells are less exhausted. (**a**) Quantification of LAG-3, TIM-3 and PD-1 on chronic stimulated human T cells under control or lactic acid conditions. n = 3 independent samples. (**b**) The gating strategy on CD8^+^CD45.1^+^ T cells from B16-OVA tumor-bearing C57BL/6N mice. (**c**) The statistical analysis of TOX in CD45.1^+^ TILs from Fig. 6F. n = 3 independent samples. (**d**) Representative histograms and statistical analysis of TOX in chronic stimulated human T cells cultured in control or lactic acid conditions. n = 3 independent samples. (**e**) Left: Representative FACS plots of LY108 and TIM-3 on CD45.1^+^ TILs from B16-OVA tumor-bearing C57BL/6N mice (n = 5 mice). Right: The statistical percentage of LY108^+^TIM-3^-^ population. (**f**) After stimulating 4.5 h with PMA in the presence of BFA, the expression of IFN-γ and TNF-α in CD45.1^+^ TILs from B16-OVA tumor-bearing C57BL/6N mice were determined by flow cytometry (n = 5 mice). Left: Representative FACS plots of IFN-γ and TNF-α. Right: The statistical percentage of IFN-γ^+^TNF-α^+^ population. Data are presented as mean ± SEM. Statistical analyses were determined by unpaired two-tailed Student’s *t*-test. ns, non-significant. Source data

To further explore the effects of persistent ↑[H^+^] exposure on T cell exhaustion in vitro, we measured LAG-3 and TIM-3 expression in ↑[H^+^]-primed human T cells that underwent multiple rounds of further stimulation with anti-CD3 antibodies and were conditioned in control medium (Fig. 6a). We found both LAG-3 and TIM-3 expression were reduced in ↑[H^+^]-exposed T cells (Fig. 6b and Extended Data Fig. 10a), indicating that ↑[H^+^] treatment results in reduced T cell exhaustion. Furthermore, we found that a high concentration of methionine leads to an increase in expression of inhibitory markers, such as PD-1, LAG-3 and TIM-3, whereas treatment with a low methionine concentration slightly decreased expression of these markers (Supplementary Fig. 6a–c). Notably, upon long-term in vitro ↑[H^+^] exposure, the frequency of TIM-3^+^LAG-3^+^ infiltrating OT-I T cells and CD19-CAR T cells within the tumors was largely reduced after adoptive transfer (Fig. 6c–e and Extended Data Fig. 10b), which is consistent with their lower exhaustion in vitro and improved tumor control capacity in vivo. Furthermore, we demonstrated that long-term ↑[H^+^]-conditioned T cells showed significantly reduced TOX expression both in vitro and in vivo (Fig. 6f,g and Extended Data Fig. 10c,d). As described previously, exhausted T cells can be generally divided into a progenitor exhausted or terminally exhausted subset, as determined by TCF1 and TIM-3 expression^52,53^. As such, we measured the expression pattern of TCF1 and TIM-3 in CD45.1^+^ TILs and noticed that the frequency of TCF1^−^TIM-3^+^ terminally exhausted T cells was largely reduced, with a significantly increased percentage of TCF1^+^TIM-3^–^ progenitor T cells under ↑[H^+^] conditioning (Fig. 6h). Moreover, we demonstrated an increased LY108^+^TIM-3^–^ progenitor T cell population (Extended Data Fig. 10e) as well as an increased frequency of IFN-γ^+^TNF-α^+^ T cells (Extended Data Fig. 10f), further supporting the fact that long-term in vitro ↑[H^+^]-expanded adoptively transferred T cells limit exhaustion and preserve stemness.

## Discussion

Tumor-specific ACT is a promising approach for treating individuals with refractory tumors, but the therapeutic effects are largely restricted by T cell dysfunction in the TME. Recently, considerable attention has been paid to pre-conditioning T cells with transient glucose starvation, glutamine restriction, or elevated extracellular potassium (↑[K^+^]) during in vitro culture to preserve T cell stemness and improve therapeutic outcome^54–56^. In this study, we demonstrated that cultivation of CD8^+^ T cells under high levels of [H^+^] during priming surprisingly promoted the acquisition of T cell stemness and increased resistance to T cell exhaustion, greatly improving the anti-tumor effects of adoptively transferred T cells.

Excessive lactic acid accumulation within the TME has long been regarded as a key factor in tumor immune escape. For instance, lactic acid and the acidic TME have been demonstrated to suppress CD8^+^ T cell cytolytic activity and cytokine production^31–33,57^. In line with these findings, we also found that short-term exposure to ↑[H^+^] in vitro was enough to profoundly inhibit effector cytokine production but had no effect on TCF1 expression or stemness-associated metabolic features. Yet long-term ↑[H^+^] treatment unexpectedly promoted TCF1 expression and maintained the phenotype of stem-like T cells through metabolic reprogramming and epigenetic remodeling. Exposure of T cells to ↑[H^+^] after their full activation can also induce a stem-like phenotype, thus excluding the possibility that T cells exposed to low pH have simply become refractory to stimulation, rather than having adopted a stem-like state. The physiological lactic acid concentration in the blood of healthy individuals is around 1.5–3.0 mM, but it can raise to 10 mM to 40 mM in the TME^35,58^. Using a persistent anti-CD3/CD28 stimulation model, Feng et al. have recently reported that a high concentration of sodium lactate increases the H3K27ac levels at the *TCF7* super-enhancer locus through the inhibition of histone deacetylase activity, leading to increased TCF1 expression and the promotion of CD8^+^ T cell stemness^59^. In contrast, we found that long-term exposure to a relatively low concentration of lactic acid (10 mM), but not sodium lactate, can readily trigger a stem-like signature of T cells through the restriction of methionine metabolism and the regulation of H3K27me3 deposition at stemness-associated gene loci, which clearly supports distinct epigenetic regulation by sodium lactate and lactic acid. However, low-acidity (pH 6.9) treatment does not have a significant impact on TCF1 expression under persistent anti-CD3/CD28 stimulation, suggesting that the stem-like signatures of CD8^+^ T cells induced by ↑[H^+^] exposure may be overridden by TCR stimulation. TCR activation can enhance methionine uptake and reprogram methionine metabolism to assist T cell activation^19,60^. We speculated that TCR stimulation may reinforce the uptake of methionine in ↑[H^+^]-treated T cells, which had restricted methionine metabolism, therefore disturbing ↑[H^+^]-induced epigenetic stemness. In addition, T cells could utilize lactate ions as an extracellular carbon source to facilitate the acquisition of stemness in the presence or absence of persistent TCR stimulation.

Metabolic activity is closely associated with T cell differentiation, and certain metabolic interventions may greatly promote long-lived memory-T cell formation^21,37,61,62^. Accordingly, our metabolomics and isotope-tracing analyses support the notion that long-term ↑[H^+^] exposure leads to striking metabolic reprogramming, such as reduced uptake of glucose and decreased glycolysis and TCA cycle metabolism. Several studies have shown that CD8^+^ T memory cells utilize FAO to fulfill their energy needs, although this is likely an adaptation for the memory lineage differentiation, and overexpression of Cpt1α promotes T cell longevity^21,22,63,64^. However, in their model of T cell-specific genetic deletion of *Cpt1a*, Raud et al. recently demonstrated that LC-FAO is largely dispensable for T cell activation and formation of CD8^+^ T memory cells^65^. It remains a challenge to explain such a discrepancy. In the context of our study, we speculate that the reprogramming of fatty acid metabolism driven by extracellular acidosis is likely associated with the generation of memory T cells, although the precise mechanism underlying this hypothesis requires further investigations. Furthermore, we found that ↑[H^+^] exposure inhibited mTOR activity, which may be induced by glycolytic inhibition. Notably, suppression of mTOR signaling may also contribute to the metabolic shift from glycolysis to mitochondrial respiration. Indeed, these ↑[H^+^]-expanded T cells exhibited improved mitochondrial fitness, as evidenced by marked increased SRC and mitochondrial mass and decreased Δψm. These mitochondrial signatures, which are enriched in stem-like T cells and are related to longevity and superior anti-tumor activity in vivo, are closely modulated by mitochondrial fusion and fission dynamics^22,48,50^. Mitochondrial inner membrane fusion protein OPA1 plays an indispensable role in memory-T cell generation^50^. In accordance, we found that extracellular acidosis also promoted OPA1 expression in T cells, eventually resulting in the morphology equilibrium toward fusion in T cells.

Many cellular metabolites have been shown to directly contribute to epigenetic modifications. For example, one-carbon metabolism can generate the universal methyl donor SAM for histone methylation^66^. Metabolites, such as *S*-2-hydroxyglutarate and β-hydroxybutyrate, have been reported to function as epigenetic regulators of memory-T cell differentiation^67,68^. Our results showed that persistent ↑[H^+^] treatment limits methionine uptake and methionine metabolism with decreased intracellular methionine, SAM and SAH through inhibition of the expression of methionine transporters. These results may partially explain why T cells are not capable of outcompeting tumor cells for methionine uptake in the TME^69^. Mechanistically, we demonstrated that ↑[H^+^] exposure dramatically reduced MYC expression and its binding to the *SLC7A5* promoter, consequently resulting in decreased SLC7A5 expression and impaired methionine metabolism in T cells. Intriguingly, methionine supplementation can restore the expression of MYC and SLC7A5, as well as the flux of the methionine cycle in ↑[H^+^]-exposed T cells, suggesting that the restoration of methionine-uptake ability was likely due to upregulation of the methionine transporter SLC7A5 in a MYC-dependent manner. mTOR activity has been reported to regulate MYC translation^70^. Accordingly, we speculated that ↑[H^+^]-induced downregulation of MYC is likely due to translational repression through the progressive inhibition of mTOR and the impaired uptake of methionine. Such repression might be reversed by exogenous methionine supplementation, although the precise mechanism needs further study. SAM derived from the methionine cycle has been considered to mediate histone methylation and epigenetic remodeling during effector-T cell differentiation^8,71^. Consistent with the decrease of intracellular SAM in ↑[H^+^]-expanded T cells, we found that the total abundance of H3K27me3 and its occupancy at the promoter of T cell-stemness loci were both greatly reduced. H3K27me3 and H3K4me3 are selectively modified in a manner that correlates with the expression of genes central to the effector or stemness program of T cells^14^. Indeed, there was a lower enrichment of H3K4me3 within effector-gene promoters in ↑[H^+^]-expanded T cells. Thus, the reduced levels of methyl-donor SAM resulting from impaired methionine uptake and metabolism under the ↑[H^+^] condition lead to epigenetic changes that ultimately favor T cell differentiation into memory status. Interestingly, the addition of methionine under ↑[H^+^] exposure not only rescues the expression of MYC and SLC7A5 in ↑[H^+^]-exposed T cells, but also restores the total level of H3K27me3 expression, supporting an important role for the MYC–SLC7A5–methionine metabolism axis in regulating ↑[H^+^]-induced epigenetic stemness. Indeed, we found that overexpression of SLC7A5 partially impaired the ↑[H^+^]-induced stemness-like phenotype, further supporting the critical role of the methionine transporter SLC7A5 in the acquisition of the T cell memory-like phenotype under ↑[H^+^] exposure.

Most TILs have historically been thought to be exhausted owing to continual TCR exposure within tumors, but paradoxically a small subset of T cell clonotypes in TILs expressing the transcription factor TCF1 retain stem-cell-like properties. Our findings revealed that ↑[H^+^] exposure offsets T cell effector function and preserves T cell stemness via the MYC–SLC7A5–methionine metabolism axis, which provides a possible explanation for the current paradox as to why a small fraction of TILs still harbor stem-like memory or precursor properties. In fact, similar findings have been reported whereby potassium ions in the TME serve a dual role influencing both T cell effector function and stemness^56,72^, further supporting the complex effects of TME immunosuppressive factors (for example, chronic TCR stimulation, hypoxia and low glucose) on T cell function and differentiation. In addition, the acidic regions within tumors are highly dynamic and both spatially and temporally regulated, and the fitness of T cells adapted to extracellular acidosis is likely overshadowed when they encounter other harsh TME factors that can affect intratumoral T cell effector function and differentiation. In addition, we observed higher expression of MYC in terminally-exhausted T cells (LY108^−^TIM-3^+^) than in progenitor-exhausted T cells (LY108^+^TIM-3^–^), which is in line with previous reports that MYC^lo^ T cells preferentially acquire the memory fate^73^. Consistent with decreased SLC7A5 expression and methionine uptake in ↑[H^+^]-exposed T cells, the expression of SLC7A5 was also significantly reduced in the LY108^+^TIM-3^–^ progenitor-exhausted T cell population within tumors, suggesting that the MYC–SLC7A5–methionine regulatory axis may also operate in vivo. It is now apparent that less differentiated stem-like memory T cells have superior anti-tumor therapeutic effects owing to their long-term persistence and resistance to the development of dysfunction^51,74^. We herein have provided compelling evidence that long-term ↑[H^+^]-conditioned CD8^+^ T cells showed enhanced persistence and superior anti-tumor ability in vivo, with a reduced terminally-exhausted T cell population (TCF1^−^TIM-3^+^) and increased stem-like progenitor subset (TCF1^+^TIM-3^–^) within tumors. In conclusion, our present study provides insights into the multidimensional effects of ↑[H^+^] exposure on the suppression of T cell effector function and its coincident influence on T cell stemness.

## Methods

### Mice and cell lines

All animal experiments were performed with the approval of the Institutional Animal Care and Use Committee (IACUC) of Suzhou Institute of Systems Medicine (ISM-IACUC-0151-R), and animals were housed in specific-pathogen-free mouse facilities. Mice were housed in standard conditions, with 12-h/12-h light/dark cycles and a controlled temperature of 22–24 °C and humidity of 60%, with unrestricted food and water availability, and were examined daily. All tumor burdens did not exceed the permission that approved by the Institutional Animal Care and Use Committee of Suzhou Institute of Systems Medicine. CD45.1^+^ female OT-I TCR transgenic mice on a C57BL/6N background were housed together. CD45.2^+^ female C57BL/6N mice aged 6–8 weeks were purchased from the Vital River as recipients. Female NCG (NOD/ShiLtJGpt-Prkdc^em26Cd52^Il2rg^em26Cd22^/Gpt) mice aged 6–8 weeks were purchased from GemPharmatech. For one independent experiment in vivo, the CD45.1^+^ and CD45.2^+^ mice (6–12 weeks) were sex-matched. HEK-293T cells from the American Type Culture Collection (ATCC) were maintained in DMEM medium supplemented with 10% fetal bovine serum (FBS) and 1% penicillin–streptomycin. The mouse melanoma cell line-B16, from ATCC, was transduced to express OVA and maintained in DMEM medium supplemented with 10% FBS and 1% penicillin–streptomycin. K562 cells from ATCC were transduced to express human CD19 and cultured in RPMI 1640 medium supplemented with 10% FBS, 1% penicillin–streptomycin, 1% GlutaMAX, 0.1 M HEPES, 1% non-essential amino acids, 1 mM sodium pyruvate and 50 μM β-mercaptoethanol. All the above reagents were purchased from Gibco.

### Primary T cell isolation, activation and retroviral transduction

Mouse CD8^+^ T lymphocytes were isolated from the spleens of 6–12 weeks male or female OT-I mice by using CD8 naive T cell isolation kit (BioLegend) and cultured in RPMI-1640 medium supplemented with 10% FBS, 1% penicillin–streptomycin, 1% non-essential amino acids, 1% GlutaMAX, 1 mM sodium pyruvate, 0.1 M HEPES and 50 μM β-mercaptoethanol in the presence of mouse IL-2 (20U/ml, Peprotech). For one independent experiment in vitro, the male and female mice (6–12 weeks) used were sex-matched. Purified cells were activated by anti-mouse CD3 (2 μg/ml, BioLegend) and anti-mouse CD28 (1 μg/ml, BioLegend) antibodies for 48 h. Human peripheral blood mononuclear cells (PBMCs) from healthy donors were purchased from Sailybio and cultured in RMPI-1640 medium supplemented with 5% Human Serum AB (Gemini), 1% penicillin–streptomycin, 1% non-essential amino acids, 1% GlutaMAX, 1 mM sodium pyruvate, 0.1 M HEPES and 50 μM β-mercaptoethanol in the presence of human IL-2 (100 U/ml, Peprotech). Anti-human CD3 (1 μg/ml, BioLegend) and anti-human CD28 (1 μg/ml, BioLegend) monoclonal antibodies were used to activate PBMCs for 3 days. Purified mouse and human T cells were activated and expanded in the indicated culture conditions: pH 7.4, pH 6.6, lactic acid (Sangon) or sodium lactate (Sangon). The following metabolites were used to supplement pH 6.6 or lactic acid (10 mM) medium: methionine 800 μM (MCE), SAM 100 μΜ (MCE) or SAH 100 μM (Sigma). For viral transduction, 1 × 10^6^ PBMCs were activated per well in 24-well plates. After 48 h of activation, the majority of the medium was replaced with unconcentrated retroviral supernatant with 10 μg/ml polybrene (Santa Cruz). Following centrifugation at 600 g for 90 min at 30 °C, cells were cultured in the incubator for 24 h with fresh medium. The transduction was repeated 24 h later and then returned to fresh medium for culture. Low-affinity nerve growth factor receptor (LNGFR) was used to quantify the infection efficiency.

### Flow cytometry

Cells were stained with fluorescent antibodies and then analyzed by flow cytometry. For surface marker staining, cells were stained with fluorescently conjugated antibodies and Live/Dead Fixable Dead Cell Stain Kit (Invitrogen) in FACS buffer (phosphate-buffered saline (PBS) with 2% FBS), then fixed with 2% paraformaldehyde (Casmart) for 20 min at room temperature. For intracellular staining of phospho-proteins, pre-stained cells were fixed with Fixation Buffer (BioLegend) and then stained with phospho-specific antibodies in Permeabilization Buffer (Invitrogen). For detection of intracellular cytokines, cells were stimulated with phorbol myristate acetate (PMA) in the presence of Brefeldin A (BFA) (BioLegend) for 4.5 h. Then, the pre-stained cells were fixed and stained with cytokines antibodies in permeabilization buffer. For intracellular transcriptional-factor staining, cells were pre-stained with Live/Dead Fixable Dead Cell Stain Kit and fluorescent conjugated antibodies in FACS buffer to detect surface markers. The cells were then fixed for 30 min on ice using FOXP3/Transcription Factor Fixation Buffer (Invitrogen) and stained with transcription factor antibodies in Permeabilization Buffer. After staining, cells were resuspended in FACS buffer for flow cytometry. Flow cytometry data were collected by BD LSR Fortessa and BD FACSDiva (v8.0.2), and analyzed with FlowJo (v10.4) software. Representative gating strategies are provided in Extended Data Figure 10b.

### RNA sequencing

RNA-seq analysis was performed using 12-day-expanded T cells cultured in the indicated conditions as shown in Fig. 1a. Briefly, 12 days after T cell expansion, total RNA was extracted by homogenization in TRIzol (Takara) and freezing in RNase-free tubes with liquid nitrogen. RNA integrity was evaluated using the Agilent 2100 Bioanalyzer (Agilent). The libraries were constructed using the TruSeq RNA sample prep kit (FC-122-1001, Illumina). The libraries were then sequenced using an Illumina NovaSeq 6000 (PE150) platform, and approximately 40 million paired-end reads were generated (Novogene). The raw read counts were extracted and then normalized by their library size factors using DESeq2 (v1.28.1). The regularized-logarithm (r-log) transformation was used to stabilize the variance across the samples. The GO and KEGG pathway enrichment analysis of differentially expressed genes was performed with clusterProfiler (v3.16.0). Expression heat maps were generated with the R package ‘pheatmap’ (v1.0.12). GSEA v.4.0 was used for GSEA analysis.

### CUT&Tag-seq

CUT&Tag-seq was performed as previously described^75^, using the Hyperactive Universal CUT&Tag Assay Kit for Illumina (TD903, Vazyme). Briefly, human T cells were expanded for 12 days under control conditions, lactic acid, or lactic acid with 800 μM methionine. Then, 1 × 10^5^ T cells were lysed for extraction of nucleic materials and were incubated with ConA beads at room temperature for 10 min. Cells were resuspended in 50 µL antibody buffer pre-mixed with primary antibody (anti-H3K27me3 or anti-H3K4me3) and incubated overnight at 4 °C. The samples were incubated with the secondary antibody at room temperature for 30 mins and then were co-incubated with the protein A/G-Tn5 transposase at room temperature for 1 h to disturb DNA fragmentation. Purified DNA was used for library preparation and sequenced using PE150 by illumina Nova6000 sequencer.

### CUT&Tag-seq analysis

FastQC (v0.11.4) (https://www.bioinformatics.babraham.ac.uk/projects/fastqc/) was used to check the sequencing read quality. Reads were quality trimmed to a minimum phred score of 20 using trimmomatic (v0.39) (http://www.usadellab.org/cms/?page=trimmomatic). All reads produced by CUT&Tag-seq of H3K27me3 and H3K4me3 were aligned to the hg38 human genome using Bowtie2 (v2.2.8) (https://bowtie-bio.sourceforge.net/bowtie2/index.shtml) with options: –local–very-sensitive-local–no-unal–no-mixed–no-discordant–phred33 -I 10 -X 700. The samples without spike-in DNA were normalized using the ChIPseqSpikeInFree method, which is a novel normalization method to effectively determine scaling factors for samples across various conditions and treatments^76^. For H3K27me3, peaks were called using MACS2 (v2.2.6) (https://hbctraining.github.io/Intro-to-ChIPseq/lessons/05_peak_calling_macs.html) with options ‘-g hs -f BAMPE–keep-dup all–broad–broad-cutoff 0.1’. For H3K4me3, peak calling used MACS2 with parameters ‘-g hs -q 0.01 -f BAMPE–keep-dup all’. Peaks were annotated with ChIPseeker (v1.22.1) (https://guangchuangyu.github.io/software/ChIPseeker/), and visualizations were created using deepTools (v3.5.1) (https://deeptools.readthedocs.io/en/develop/) and pyGenomeTracks (v3.7) (https://pygenometracks.readthedocs.io/en/latest/).

### qRT–PCR

Total RNA was isolated with TRIzol (Takara) and reverse transcribed into cDNA with HiScript Reverse Transcriptase (Vazyme), according to the manufacturer’s instructions. For quantitative real-time PCR, the ABI prism 7500 real-time PCR System (Thermo Fisher) and 2×SYBR Green qPCR Master Mix (Bimake) were used according to the manufacturer’s instructions. *ACTB* was used as an internal standard. The relative expression of mRNA was calculated using the 2^−ΔΔCT^ method. Primer sequences used for qPCR can be found in Supplementary Table 1.

### Western blotting

For protein expression analysis, cells were collected and washed with cold PBS, and the total protein was extracted by using 1% SDS (Sangon) on ice. Protein concentration was measured by BCA protein assay kit (Merck). Protein samples were separated by SDS–PAGE gels and then transferred to PVDF-membranes (Millipore). Membranes were blocked with 5% nonfat milk in PBS containing Tween20. After blocking, membranes were incubated with primary antibodies overnight at 4 °C and HRP-coupled secondary antibodies for 2 h at room temperature. The HRP signal was developed by electrochemiluminescence (ChemeMINI610) and collected by Sage Capture (v2.19.12). Data analysis was performed using ImageJ (v1.8.0) software.

### Mitochondrial morphology analysis

In each well of 24-well plates, PBMCs were activated by human anti-CD3 and anti-CD28 antibodies for 3 days and were cultured in RPMI-1640 medium under different conditions for 12 days. Then, 1 × 10^6^ PBMCs were collected and fixed in a pre-cooled fixation buffer (2.5% glutaraldehyde, 0.1 M phosphate buffer (PB), pH 7.4) overnight at 4 °C. After being washed with PBS three times, cells were post-fixed in 1% osmium tetroxide in PBS for 2 h, dehydrated and embedded in Spurr’s resin, according to the standard procedure. Ultrathin sections were stained with uranyl acetate and lead citrate. Mitochondrial morphology was imaged by using Hitachi HT-7800 transmission electron microscopy (TEM) (v01.20) and AMT-XR81DIR camera.

### Antibodies and reagents

Antibodies used for flow cytometric analysis were as follows. APC anti-human NGFR (cat. no. 345108, 1:1,000 for FACS), FITC anti-human CD4 (cat. no. 357406, 1:200 for FACS), Pacific Blue anti-human CD8α (cat. no. 300928, 1:200 for FACS), APC-Cy7 anti-human/mouse CD44 (cat. no. 103028, 1:200 for FACS), PE anti-human CCR7 (cat. no. 353204, 1:200 for FACS), PE anti-human TNF-α (cat. no. 502909, 1:200 for FACS), PE-Cy7 anti-human CD45RO (cat. no. 304230, 1:200 for FACS), APC anti-human IFN-γ (cat. no. 502512, 1:200 for FACS), PE-Cy7 anti-human LAG-3 (cat. no. 369310, 1:200 for FACS), PE anti-human TIM-3 (cat. no. 345006, 1:200 for FACS), BV711 anti-human PD-1 (cat. no. 329928, 1:200 for FACS), Percp-Cy5.5 anti-human CD62L (cat. no. 304824, 1:200 for FACS), APC anti-human CD27 (cat. no. 302810, 1:200 for FACS), BV711 anti-mouse CD8α (cat. no. 100748, 1:200 for FACS), PE-Cy7 anti-mouse CD62L (cat. no. 104418, 1:200 for FACS), APC-Cy7 anti-mouse CD45.2 (cat. no. 830789, 1:200 for FACS), Pacific Blue anti-mouse CD45.1 (cat. no. 110722, 1:200 for FACS), APC anti-mouse Ly108 (cat. no. 134610, 1:200 for FACS), PE anti-mouse LAG-3 (cat. no. 125207, 1:200 for FACS), Alexa Fluor 488 anti-c-MYC Antibody (cat. no. 626811, 1:200 for FACS), PE anti-mouse TNF-α (cat. no. 506306, 1:200 for FACS), and APC anti-mouse IFN-γ (cat. no. 505810, 1:200 for FACS) were purchased from BioLegend. Live/Dead Fixable Dead Cell Stain Kit, PerCP-eFluor710 anti-mouse PD-1 (cat. no. 46-9981-82, 1:200 for FACS), PE phospho-S6 (Ser^235/236^) (cat. no. 12-9007-42, 1:200 for FACS), PE anti-human/mouse TOX (cat. no. 12-6502-82, 1:200 for FACS), and PE-Cy7 anti-mouse TIM-3 (cat. no. 25-5870-82, 1:200 for FACS) were purchased from Thermo Fisher. Alexa Fluor 647 anti-TCF1 (cat. no. 6709, 1:200 for FACS) and phospho-4E-BP1 (Thr^37/46^) (cat. no. 2846, 1:200 for FACS) were purchased from Cell Signaling Technology. Alexa Fluor 647 anti-puromycin (cat. no. MABE343-AF647, 1:800 for FACS) was purchased from Merck.

Antibodies used for western blots were as follows. Rabbit anti-phospho-Akt (Ser^473^) (cat. no. 4060, 1:1,000 for IB), anti-phospho-NF-κB p65 (Ser^536^) (cat. no. 3033, 1:1,000 for IB), anti-c-MYC (cat. no. 18583, 1:1,000 for IB), anti-EZH2 (cat. no. 5246, 1:1,000 for IB), anti-tri-methyl-histone H3 (Lys27) (cat. no. 9733, 1:1,000 for IB), anti-tri-methyl-histone H3 (Lys4) (cat. no. 9751, 1:1,000 for IB), anti-di-methyl-histone H3 (Lys9) (cat. no. 4658, 1:1,000 for IB), anti-di-methyl-histone H3 (Lys79) (cat. no. 5427, 1:1,000 for IB), anti-histone H3 (cat. no. 9715, 1:1,000 for IB), anti-COXIV (cat. no. 850, 1:1,000 for IB), anti-rabbit IgG (HRP-linked) (cat. no. 7074, 1:2,000 for IB), and anti-mouse IgG (HRP-linked) (cat. no. 7076, 1:2,000 for IB) were purchased from Cell Signaling Technology. Mouse anti-Tim23 (cat. no. 611223, 1:1,000 for IB) was from BD Biosciences. Mouse anti-β-actin (cat. no. 66009-1-Ig, 1:5,000 for IB), anti-SLC7A5 (cat. no. 67951-1-Ig, 1:1,000 for IB), and rabbit anti-SLC38A1 (cat. no. 12039-1-AP, 1:1,000 for IB) were from Proteintech. Rabbit anti-SLC38A2 (cat. no. BMP081, 1:1,000 for IB) was purchased from Medical & Biolohical Laboratories.

### T cell metabolic assay

To check the BODIPY FL C_16_ uptake in control or ↑[H^+^]-treated T cells, T cells were cultured with freshly dissolved 1 μM BODIPY FL C_16_ (Invitrogen) for 1 h, then washed twice with PBS prior to surface staining. To further explore the metabolic dependence in different treatment groups, experiments were performed using the SCENITH (single cell energetic metabolism by profiling translation inhibition) method^42^. Briefly, T cells were seeded in 96-well plates at 1 × 10^6^ cells/ml. Then cells were treated with control (Ctrl), 2-deoxy-d-glucose (final concentration 100 mM), oligomycin (final concentration 5 μM), or a mix of the inhibitors at the final concentrations for 40 min at 37 °C. Puromycin (final concentration 10 μg/ml) was added during the last 30 min. After puromycin treatment, cells were washed with cold PBS and pre-stained with Live/Dead Fixable Dead Cell Stain Kit and antibodies against surface markers. The staining of intracellular puromycin was followed by the staining process for intracellular transcription factors.

### Metabolomic analysis with LC–MS/MS

Metabolomic analysis and sample collection were performed as in previous reports^77^. In brief, PBMCs were activated individually in 24-well plates by human anti-CD3/CD28 for 3 days and were cultured in RPMI 1640 medium with control or lactic acid to day 12. Then, 8 × 10^6^ cells per sample (*n* = 4 independent samples per group) were collected and transferred to a 1.5-ml tube for centrifugation at 300 g for 5 min at 4 °C and then washed with cold PBS. After centrifugation at 300 g for 5 min again, 80% cold methanol was added and vigorously vortexed to disrupt the cell pellet completely, and then the cells were transferred to a freezer at −80 °C. Samples were centrifuged at 12,000 g for 10 min at 4 °C. The supernatant was collected. Finally, the extracts were lyophilized and analyzed by UHPLC–MS/MS in Novogene. The raw data files generated by UHPLC–MS/MS were processed using the Compound Discoverer (v3.1, Thermo Fisher) to perform peak alignment, peak picking and quantitation for each metabolite. Metaboanalyst (v5.0) software was used for further data analysis, and then significantly enriched pathways were selected using *P* < 0.05.

### Stable-isotope-labeling experiments

The ^13^C metabolic flux was performed on T cells using previously described methods^60,78,79^. Briefly, PBMCs were activated per well in 24-well plates with human anti-CD3/CD28 for 3 days, as described above. For [^13^C_6_] glucose tracing, medium was switched after 11 days to glucose-free RPMI (Gibco) supplemented with 10% dialyzed FBS (Thermo Fisher Scientific), 1% penicillin–streptomycin, 1% non-essential amino acids, 50 μM β-mercaptoethanol and 0.1 M HEPES, containing 11 mM [^13^C_6_]glucose (Cambridge Isotope Laboratories) with control or 10 mM lactic acid, for 24 h. For [^13^C_16_]palmitate tracing, 2 × 10^7^ T cells were activated and placed in completed medium with control or 10 mM lactic acid conditions as described above at day 12, containing 200 μM [^13^C_16_]palmitate (Cambridge Isotope Laboratories), for 8 h. For [^13^C_5_]methionine tracing, T cells were expanded under control, lactic acid or lactic acid with 800 μM methionine conditions for 12 days. Then, 4 × 10^7^ T cells were switched to methionine-free complete RPMI medium (Gibco) and cultured under control conditions, lactic acid, or lactic acid supplemented with methionine (containing 100 μM, 100 μM, or 800 μM [^13^C_5_]methionine) (Cambridge Isotope Laboratories), for 8 h. The respective supernatants were collected and then stored at −80 °C. Cells were pelleted by centrifugation (300 g, 4 °C, 5 min), washed twice with saline and immediately flash-frozen in liquid nitrogen and stored at −80 °C. Metabolites were extracted by using ice-cold HPLC-grade 80% methanol and vortexed briefly, followed by the addition of 200 ml HPLC-grade water, then 500 ml HPLC-grade chloroform (methanol:water:chloroform ratio, 5:2:5). The mixture was vortexed and centrifuged to achieve phase separation. The supernatants were dried by vacuum spin for subsequent derivatization, and then incubated with 2% (wt/vol) methoxyamine hydrochloride (226904, Sigma-Aldrich) in pyridine for 60 min at 37 °C, and silylated by *N*-methyl-*N*-(tert-butyldimethylsilyl) trifluoroacetamide with 1% tert-butyldimethylchlorosilane (TBDMS, 18162-48-6, Regis Technologies) for 30 min at 45 °C. The [^13^C_6_]glucose derivatives were analyzed by GC-HRMS, the Trace 1310 gas chromatograph (Thermo Fisher) with the DB–35MS column (Agilent Technologies) and the Q Exactive GC Orbitrap GC–MS/MS system (Thermo Fisher). GC–MS/MS metabolomics assays were conducted by Metabo-Profile. The metabolites were identified and quantified by Xcalibur (v4.1) and TraceFinder (v5.1) (Thermo Fisher), including retention time, charge to mass ratio of ion fragments, and peak intensity. The [^13^C_16_]palmitate and [^13^C_5_]methionine derivatives were analyzed by ultrahigh-pressure liquid chromatography–triple quadrupole mass spectrometer (UPLC–TQMS). The raw data from UPLC–TQMS were analyzed using Waters’ MassLynx software (v4.1, Waters) for peak extraction, integration, identification and quantification of the metabolites. R language (v4.1.1) was used for subsequent statistical analysis.

### B16 tumor model and adoptive cell transfer immunotherapy

To investigate the anti-tumor activity of T cells in vivo, 0.2 × 10^6^ B16-OVA melanoma cells were subcutaneously injected into female C57BL/6N mice. Nine days after tumor implantation, mice were intravenously injected with 5 × 10^6^ T cells from female OT-I mice expanded for 7 days in different conditions. Tumor-bearing mice received 5 Gy of sublethal irradiation prior to ACT. Tumor area was measured every 2 days and calculated as length (mm) × width (mm). Mice with a tumor area approaching 300 mm^2^ were euthanized. To explore the persistence of expanded T cells in different conditions, 4 × 10^6^ CD45.1^+^ T cells from female OT-I mice were adoptively transferred into female C57BL/6N mice. One week later, mice were euthanized and the cells from isolated blood, spleens, and lymph nodes were counted. The proportion of transferred T cells was determined by gating on T cells expressing CD45.1^+^ and CD45.2^+^ through flow cytometry.

### NCG mice model and CD19-CAR-T therapy

Female NCG mice were implanted subcutaneously with 1 × 10^6^ CD19-K562 cells. When tumor volumes reached 75 mm^3^, mice received 5 × 10^6^ non-transduced T cells and CD19-CAR T cells cultured in control or 10 mM lactic lactic-acid-containing medium. Tumor growth was measured every 2 days with electronic calipers and calculated by length (mm) × width (mm). Mice with a tumor area larger than 300 mm^2^ were euthanized. The tumors were collected, digested and processed with Percoll (Sigma), and then the T cell function and phenotype were determined by flow cytometry.

### Basal oxygen consumption rate and extracellular acidification rate analysis

For analysis of metabolic characteristics, the OCR and ECAR were assessed by seahorse XF24 analyzer (Agilent). Briefly, human T cells cultured with different conditions for 12 days were pretreated with non-buffered XF medium (non-buffered RPMI 1640 containing 10 mM glucose and 1 mM sodium pyruvate, and 2 mM glutamine). Next, human T cells were seeded at 0.5 × 10^6^ cells per well in a XF24 cell culture microplate and incubated in non-CO_2_ incubator for 1 h at 37 °C. To enhance cell adherence, the plates were spun at room temperature for 5 min at 100 g with zero brake. Oxygen consumption and extracellular acidification were analyzed under basal conditions and in response to 1.25 μM oligomycin, 50 mM 2-deoxy-d-glucose, 1.5 μM FCCP, 0.5 μM rotenone and 0.5 μM antimycin A. SRC was calculated by subtracting the basal OCR from the maximum OCR.

### ChIP–qPCR

Chromatin immunoprecipitation (ChIP) was performed following the manufacturer’s instructions provided with the ChIP-IT express enzymatic shearing kit (Active Motif). In brief, 15 million human T cells cultured in control or 10 mM lactic acid conditions were fixed with formaldehyde for 10 min at room temperature, and the fixation reaction was stopped by the addition of 1× glycine. Fixed cells were pelleted with PMSF and protein inhibition cocktail and stored at −80 °C prior to cell lysis. Then, thawed pellet was resuspended in 1 ml ice-cold lysis buffer with PMSF and protein inhibition cocktail on ice for 30 min to obtain nuclear material. The nuclear pellet was then resuspended and digested with enzymatic cocktail to shear chromatin for 15 min at 37 °C. Sheared chromatin was incubated with protein G magnetic beads with anti-c-MYC (CST, cat. no. 18583, 1:100 for ChIP) and anti-IgG (CST, cat. no. 2729, 1:100 for ChIP) at 4 °C overnight. Then, magnetic beads were washed with ChIP buffers four times and eluted with 50 μl elution buffer. The eluted DNA was reverse crosslinked for 2.5 h at 65 °C and then purified by phenol chloroform extraction. The purified DNA was used to perform ChIP–qPCR to detect the enrichment of MYC at target genes’ promoters. The primers used for ChIP–qPCR can be found in Supplementary Table 2.

### Mitochondrial mass and membrane potential analysis

Mitochondrial mass and membrane potential were analyzed using MitoTracker Green and tetramethyrhodamine methyl ester (TMRM). Cells were stained with 250 nM MitoTracker Green (Thermo Fisher) or 50 nM TMRM (Thermo Fisher) in an incubator at 37 °C (5% CO_2_) for 1 h before cell surface staining. Cells were washed with FACS buffer three times, followed by surface markers staining for further FACS analysis.

### Statistical analysis

Statistical analysis was performed using GraphPad Prism version 8.0. Results are displayed as mean ± s.e.m. Two-tailed Student’s *t*-test was used to compare treatments with control groups. Two-way ANOVA with Tukey’s or Sidak’s or Dunnett’s multiple-comparisons test was applied for multiple comparisons. *P* < 0.05 was considered to be statistically significant.

### Reporting summary

Further information on research design is available in the Nature Portfolio Reporting Summary linked to this article.

## Supplementary information

Supplementary Information Supplementary Figures 1–6 and Supplementary Tables 1 and 2.

Reporting Summary

Supplementary Data Statistical source data for Supplementary Figures

## Data Availability

The RNA-seq raw data sets generated during this study have been deposited to NCBI GEO database under accession numbers GSE216623 and GSE219257. The CUT&Tag-seq datasets are available at the GEO (accession number GSE216623). Metabolomics data have been deposited to the EMBL-EBI MetaboLights database with the identifier MTBLS6661. Any additional materials and reagents are available from the corresponding author upon reasonable request. Source data are provided with this paper.

## Source data

Source Data Fig. 1 Statistical Source Data
Source Data Fig. 2 Statistical Source Data
Source Data Fig. 3 Statistical Source Data
Source Data Fig. 4 Statistical Source Data
Source Data Fig. 5 Statistical Source Data
Source Data Fig. 6 Statistical Source Data
Source Data Extended Data Fig. 1 Statistical Source Data
Source Data Extended Data Fig. 2 Statistical Source Data
Source Data Extended Data Fig. 3 Statistical Source Data
Source Data Extended Data Fig. 4 Statistical Source Data
Source Data Extended Data Fig. 5 Statistical Source Data
Source Data Extended Data Fig. 6 Statistical Source Data
Source Data Extended Data Fig. 7 Statistical Source Data
Source Data Extended Data Fig. 8 Statistical Source Data
Source Data Extended Data Fig. 9 Statistical Source Data
Source Data Extended Data Fig. 10 Statistical Source Data
Source data unprocessed western blots Unprocessed western blots

## Code availability

The codes used in this article have been deposited at https://github.com/LuChenLab/T_cells_stemness.
