# Supplementary material for: Extracellular acidosis restricts one-carbon metabolism and preserves T cell stemness
Source: Nat Metab. 2023 Jan 30;5(2):314–30. doi: 10.1038/s42255-022-00730-6 (PMC9970874; doi:10.1038/s42255-022-00730-6)
Supplement: Supplementary file 1 — Supplementary Figures 1–6 and Supplementary Tables 1 and 2. [file 42255_2022_730_MOESM1_ESM.pdf]

nature metabolism

Article

https://doi.org/10.1038/s42255-022-00730-6

Extracellular acidosis restricts one-carbon metabolism and preserves T cell stemness

In the format provided by the authors and unedited

Supplementary Figure 1

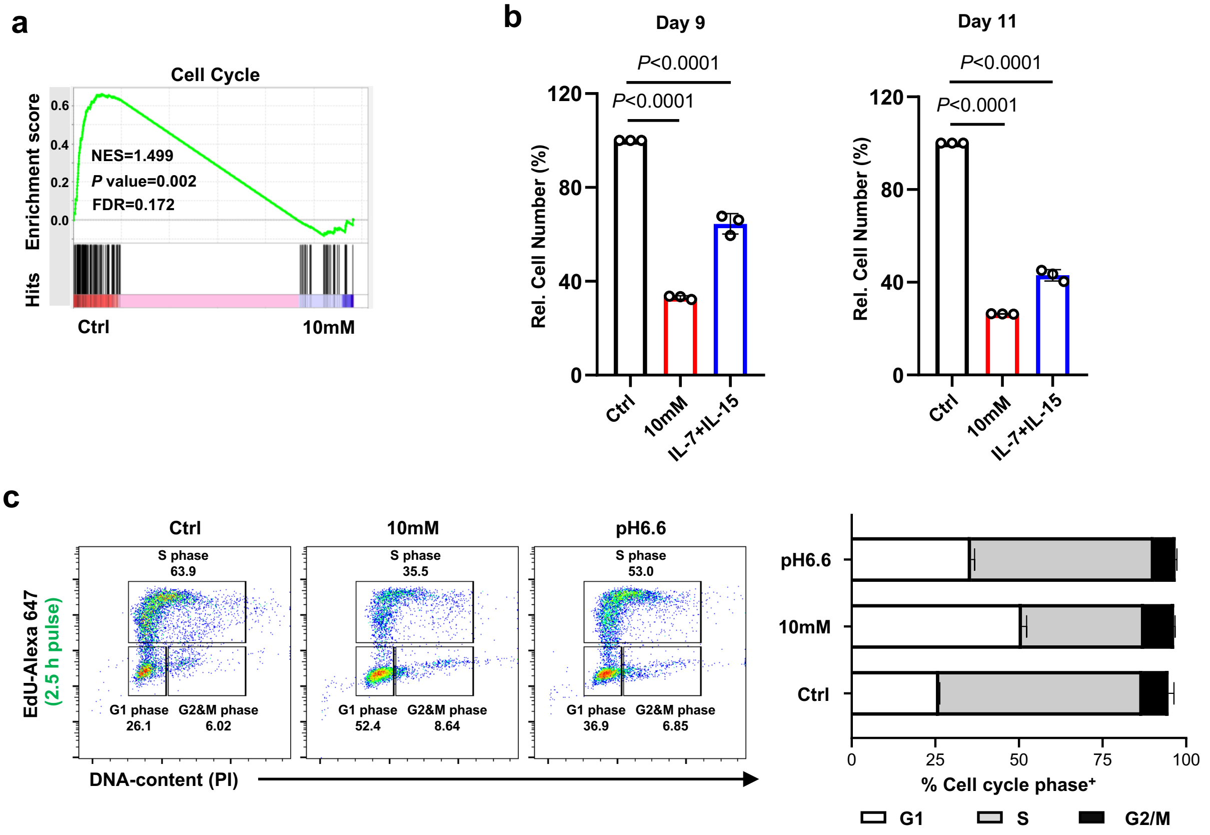

Supplementary Fig. 1 Elevated [H+] inhibits T cell proliferation. (**a**) GSEA plot of the cell cycle pathway in T cells expanded for 12 days under long-term lactic acid treatment versus control. (**b**) Pre-activated human CD8+ T cells were rested and cultured with the indicated conditions for 9 or 11 days, and cell density was counted. n = 3 independent samples. (**c**) Left: Representative dot plots showed EdU/DNA profiles of pre-activated human CD8+ T cells after ↑[H+] exposure for 5 days. Right: Bar graph depicts the percentage of T cells in the indicated cell cycle phases under different treatments. n = 3 independent samples. Data are presented as mean ± SEM. Nominal *P* value and FDR were calculated with default method of GSEA software (**a**). Statistical analyses were determined by unpaired two-tailed Student's *t*-test (**b**).

Supplementary Figure 2

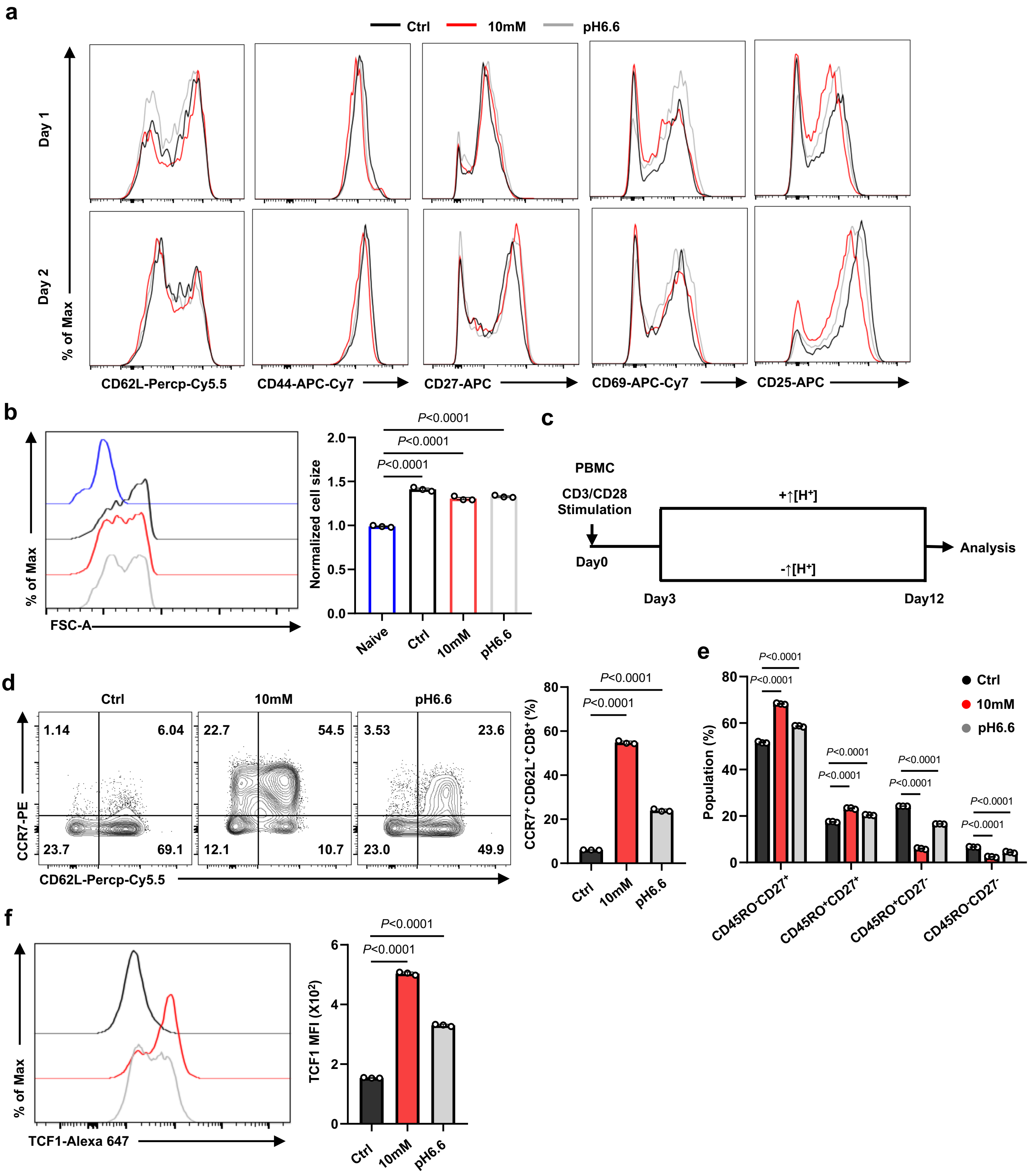

Supplementary Fig. 2 ↑[H^+] treatment has slight effects on T cell activation and induces a stemness-like phenotype after T cell full activation. (**a**) Expression of surface markers (CD62L, CD44, CD27, CD69, CD25) in human $CD8^+$ T cells activated by anti-CD3 and anti-CD28 for 2 days in control, lactic acid or pH6.6 conditions. (**b**) Flow cytometric analysis and quantification of forward scatter (FSC) in naïve and activated human $CD8^+$ T cells on day 2 after anti-CD3 and anti-CD28 stimulation. n = 3 independent samples. (**c**) Schematic representation of T cell full activation for 3 days and then cultured in the acidic or non-acidic conditions, for 9 days. (**d-f**) Representative expression of CCR7/CD62L (**d**), CD45RO/CD27 (**e**), and TCF1 (**f**) in human $CD8^+$ T cells under treatments as shown in (**c**). n = 3 independent samples. Data are presented as mean ± SEM. Statistical analyses were determined by unpaired two-tailed Student's *t*-test (**d, f**), two-way ANOVA with Tukey's multiple-comparisons test (**b**) or two-way ANOVA with Dunnett's multiple-comparisons test (**e**).

Supplementary Figure 3

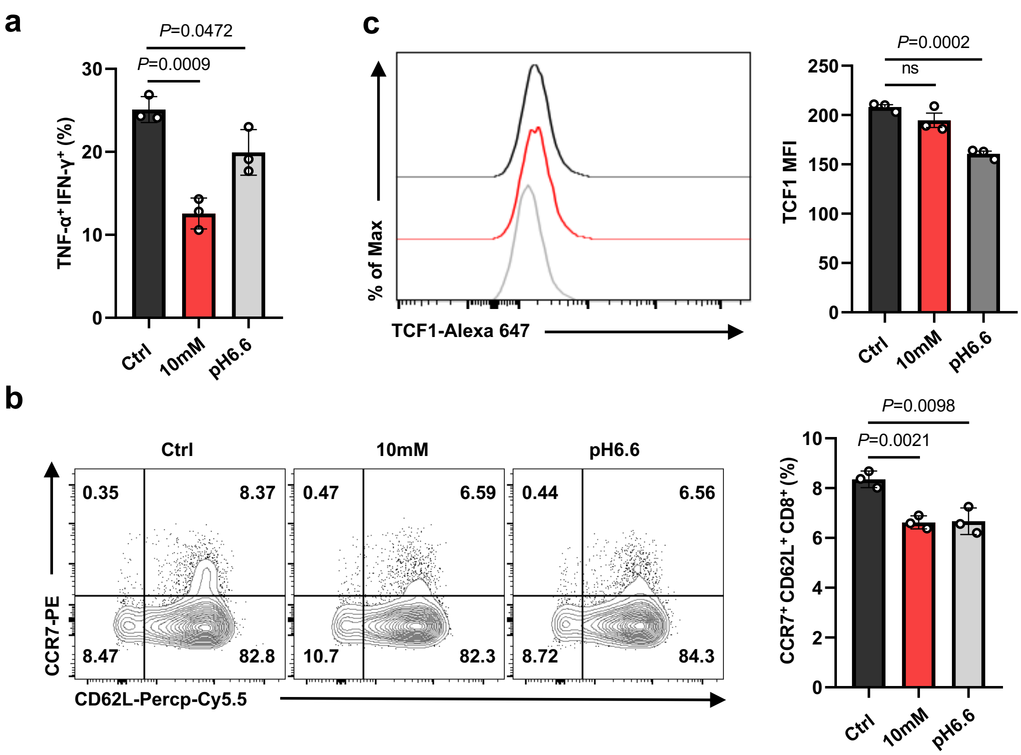

Supplementary Fig. 3 Short-term ↑[H$^+$] exposure has no effects on stem-like signatures. (**a**) Statistics of cytokines expression in human CD8$^+$ T cells activated for 11 days and then cultured with control, lactic acid, and pH6.6 conditions for 24 h. n = 3 independent samples. (**b**) Representative plots and quantification of CCR7$^+$CD62L$^+$ population in human CD8$^+$ T cells treated as in (**a**). n = 3 independent samples. (**c**) Representative histograms and quantification of TCF1 expression in human CD8$^+$ T cells treated as in (**a**). n = 3 independent samples. Data are presented as mean ± SEM. Statistical analyses were determined by unpaired two-tailed Student's *t*-test. ns, non-significant.

Supplementary Figure 4

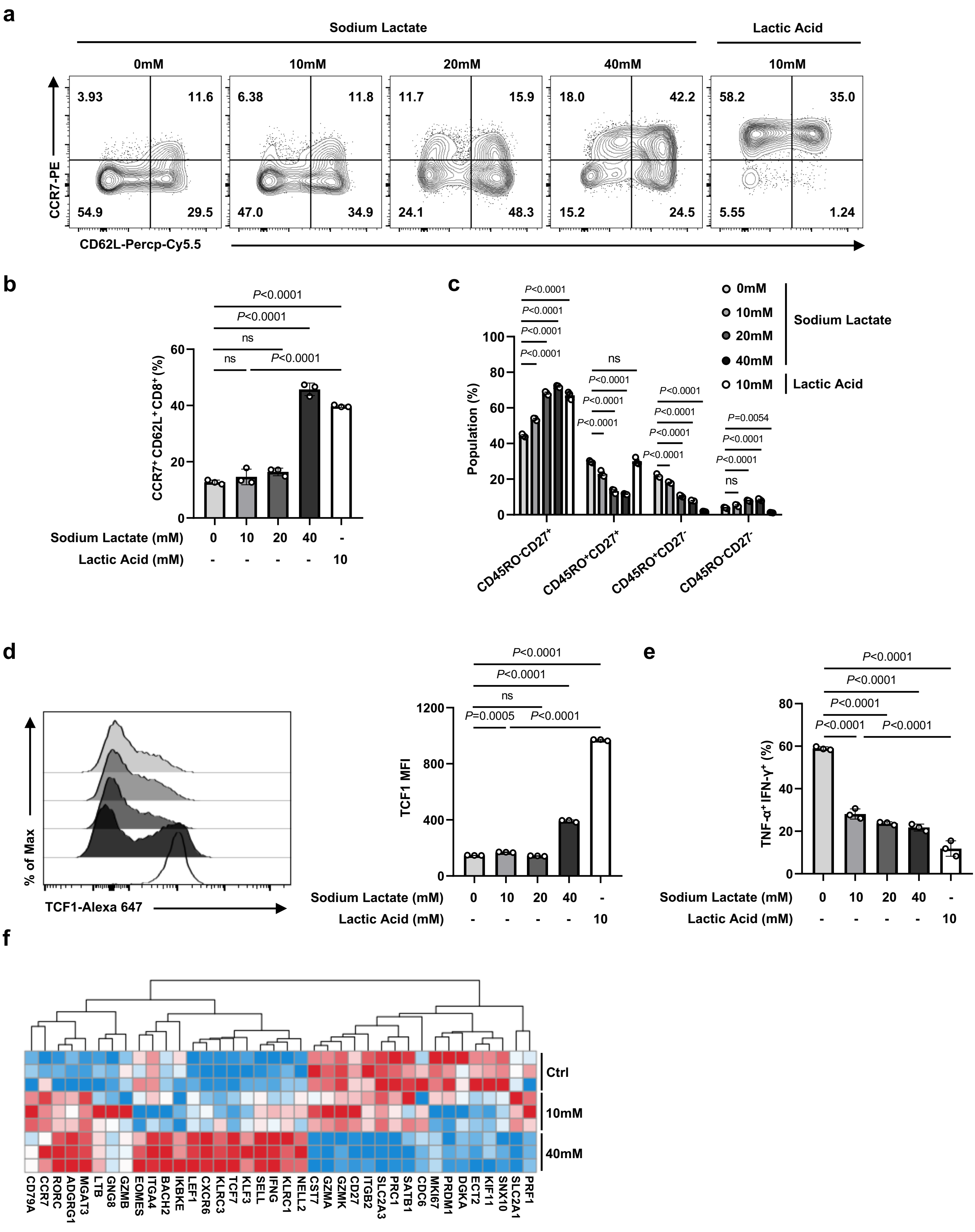

Supplementary Fig. 4 Extracellular acidosis shows much higher efficacy in promoting a T cell stemness-like state as compared to sodium lactate. (**a-e**) Human T cells were activated individually in 24-well plates by human anti-CD3/CD28 and expanded for 12 days under control, lactic acid (10mM), or different sodium lactate (10mM, 20mM, and 40mM) conditions. Representative expression of CCR7/CD62L (**a, b**), CD45RO/CD27 (**c**), TCF1 (**d**), and IFN-γ/TNF-α (**e**) in human $CD8^{+}$ T cells cultured with indicated conditions. n = 3 independent samples. (**f**) Human T cells were activated by human anti-CD3/CD28 and expanded for 12 days under control, sodium lactate (10mM), or sodium lactate (40mM) conditions, and the collected the cells for RNA-seq analysis. Heat map of selected genes associated with memory and effector T cells. Data are presented as mean ± SEM. Statistical analyses were determined by two-way ANOVA with Tukey's multiple-comparisons test (**b, d, e**) or two-way ANOVA with Dunnett's multiple-comparisons test (**c**). ns, non-significant.

Supplementary Figure 5

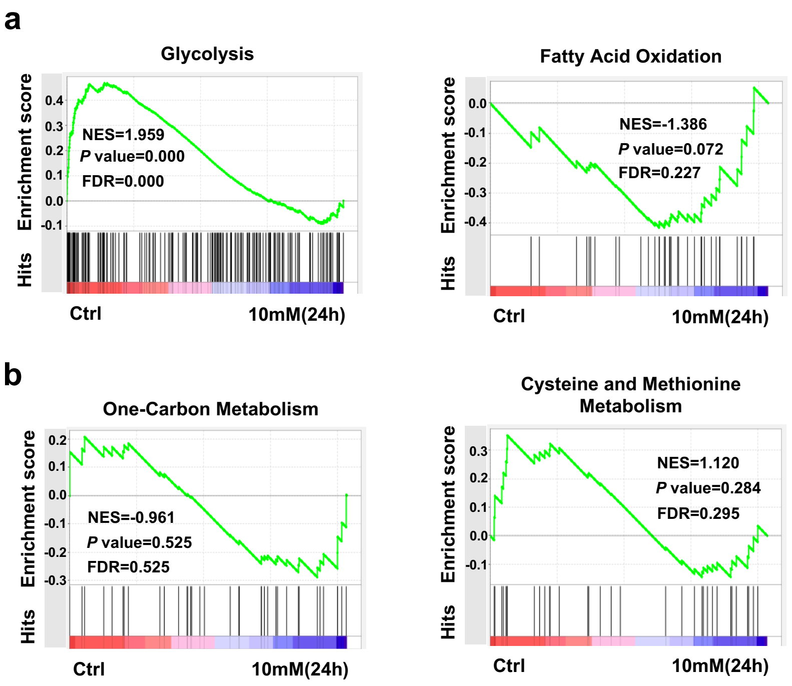

Supplementary Fig. 5 The effects of short-term ↑[H$^+$] exposure on T cell metabolism. Human T cells were activated individually in 24-well plates by human anti-CD3/CD28 and expanded in completed medium for 11 days, and then cultured in control or lactic acid (10mM) for 24 h. GSEA plots comparing enrichment of glycolysis and fatty acid metabolism (**a**). GSEA plots comparing enrichment of one-carbon metabolism and cysteine and methionine metabolism (**b**). Nominal *P* value and FDR were calculated with default method of GSEA software.

Supplementary Figure 6

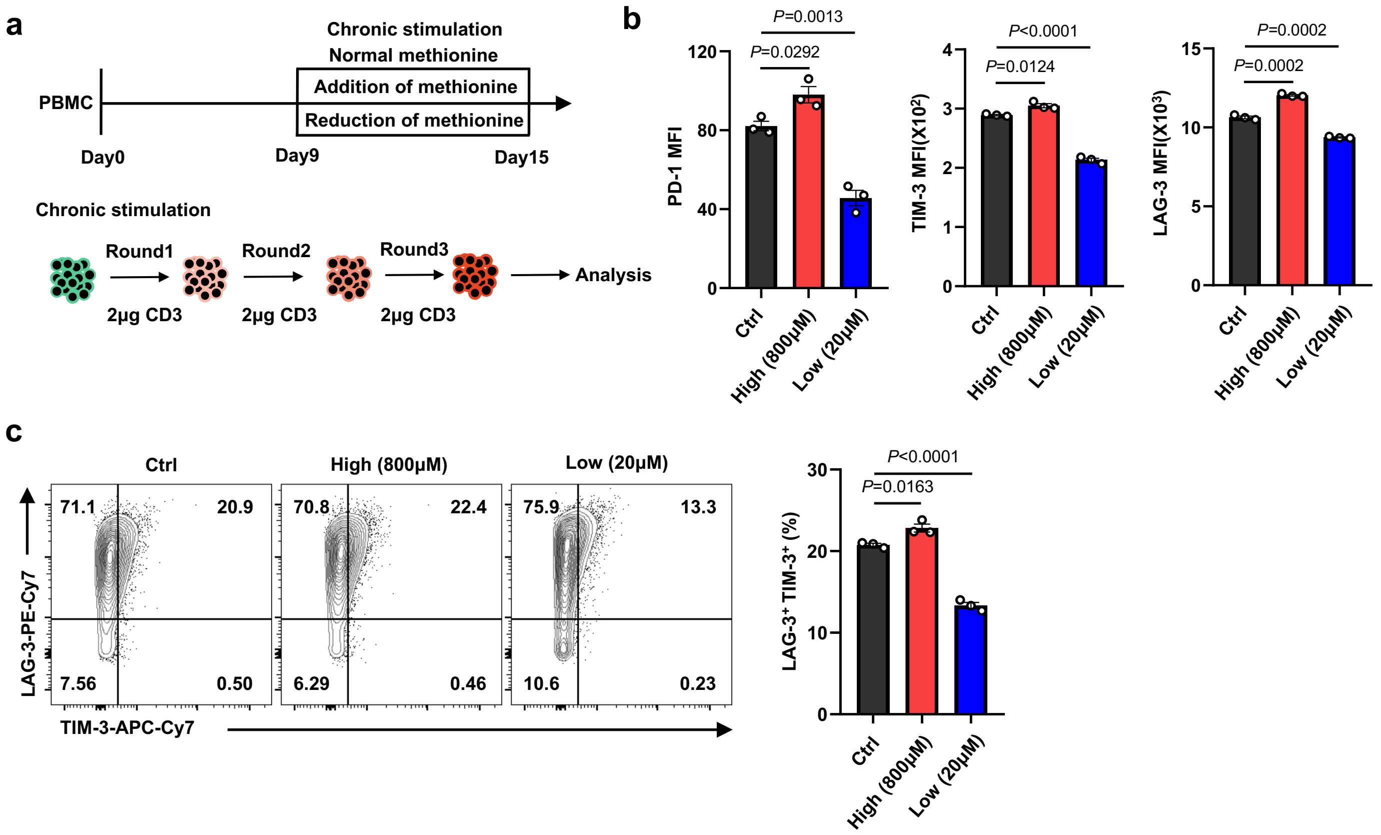

Supplementary Fig. 6 The effects of methionine on T cell exhaustion *in vitro*. (**a**) The schematic of chronic stimulation of human T cells *in vitro*. In brief, human T cells were expanded in normal conditions for 9 days, and then cultured in normal (100µM), high (800µM) methionine or low (20µM) methionine conditions with anti-CD3 for 6 days. (**b**) Effects of methionine on the expression of exhausted markers (PD-1, TIM-3, LAG-3) during chronic stimulation *in vitro*. n = 3 independent samples. (**c**) Representative FACS plots (Left) and quantification (Right) for TIM-3^{+}LAG-3^{+}CD8^{+} populations in chronically stimulated T cells as shown in (**a**). n = 3 independent samples. Data are presented as mean ± SEM. Statistical analyses were determined by unpaired two-tailed Student's *t*-test.

Supplementary Table 1. Primer for qRT-PCR.

Gene name	Forward primer	Reverse primer
ACTB (H)	CATGTACGTTGCTATCCAGGC	CTCCTTAATGTCACGCACGAT
BACH2 (H)	AATACCAGCTTGCATGTACCAA	TTATCTTCCCGGAATGTGCTTG
TCF7 (H)	CTGGCTTCTACTCCCTGACCT	ACCAGAACCTAGCATCAAGGA
KLF2 (H)	CTACACCAAGAGTTCGCATCTG	CCGTGTGCTTTCGGTAGTG
LEF1 (H)	AGAACACCCCGATGACGGA	GGCATCATTATGTACCCGGAAT
OPA1 (H)	TGTGAGGTCTGCCAGTCTTTA	TGTCCTTAATTGGGGTCGTTG
MFN1 (H)	ATGACCTGGTGTTAGTAGACAGT	AGACATCAGCATCTAGGCAAAAC
MFN2 (H)	CTCTCGATGCAACTCTATCGTC	TCCTGTACGTGTCTTCAAGGAA
LDHA (H)	ATGGCAACTCTAAAGGATCAGC	CCAACCCCAACAACTGTAATCT
CPT1α (H)	ATGCGCTACTCCCTGAAAGTG	GTGGCACGACTCATCTTGC
SLC2A1 (H)	ATTGGCTCCGGTATCGTCAAC	GCTCAGATAGGACATCCAGGGTA
SLC2A3 (H)	GCTGGGCATCGTTGTTGGA	GCACTTTGTAGGATAGCAGGAAG
SLC7A5 (M)	GGTCTCTGTTCACGTCCTCAAG	GAACACCAGTGATGGCACAGGT
MTR (H)	CGCAACCCGAAGGTCTGAA	TTCTTCGTTTAGCTTCTCCCG
AHCY (H)	ATCCTTGGCCGGCACTTTGAG	TCCACCTGCGGCTTGATGTTC
BHMT (H)	CTGTGTGGGCAGTTGAAACC	TGCTGCTCAGTTGTGGCTTC
SHMT1 (H)	CAGGGCTCTGTCTGATGCAC	CGTAACGCGCTCTTGTCAC
SHMT2 (H)	GCGGATGTTGTTACCACC	GGGAACACAGCGAAGTTGAT

Supplementary Table 2. Primer for ChIP-PCR.

Gene name	Forward primer	Reverse primer
NCL	CTCGGGGTGGAGAGATGAGA	GACTCCGACTAGGGCCGATA
SLC7A5	GTCTCCATGGCGCAGGAG	CGCAGCTGCGTCAGGAAC
SLC38A1	AGTAAGCGGGAAAAAAGGGA	ATTTGCTGAGCACCGAGAAA
SLC38A2	TAGGGAACACAGGAGTGTGA	CTACTGCTCTCTTCCTTTTG
