# Supplementary material for: Extracellular acidosis restricts one-carbon metabolism and preserves T cell stemness
Source: Nat Metab. 2023 Jan 30;5(2):314–30. doi: 10.1038/s42255-022-00730-6 (PMC9970874; doi:10.1038/s42255-022-00730-6)
Supplement: Supplementary file 2 — Reporting Summary [file 42255_2022_730_MOESM2_ESM.pdf]

nature portfolio

Corresponding author(s): Lu Chen, Lianjun Zhang, Guideng Li

Last updated by author(s): Dec 16, 2022

Reporting Summary

Nature Portfolio wishes to improve the reproducibility of the work that we publish. This form provides structure for consistency and transparency in reporting. For further information on Nature Portfolio policies, see our Editorial Policies and the Editorial Policy Checklist.

Statistics

For all statistical analyses, confirm that the following items are present in the figure legend, table legend, main text, or Methods section.

n/a	Confirmed	
☐	☒	The exact sample size (n) for each experimental group/condition, given as a discrete number and unit of measurement
☐	☒	A statement on whether measurements were taken from distinct samples or whether the same sample was measured repeatedly
☐	☒	The statistical test(s) used AND whether they are one- or two-sided<br>*Only common tests should be described solely by name; describe more complex techniques in the Methods section.*
☒	☐	A description of all covariates tested
☐	☒	A description of any assumptions or corrections, such as tests of normality and adjustment for multiple comparisons
☐	☒	A full description of the statistical parameters including central tendency (e.g. means) or other basic estimates (e.g. regression coefficient) AND variation (e.g. standard deviation) or associated estimates of uncertainty (e.g. confidence intervals)
☐	☒	For null hypothesis testing, the test statistic (e.g. F, t, r) with confidence intervals, effect sizes, degrees of freedom and P value noted<br>*Give P values as exact values whenever suitable.*
☒	☐	For Bayesian analysis, information on the choice of priors and Markov chain Monte Carlo settings
☒	☐	For hierarchical and complex designs, identification of the appropriate level for tests and full reporting of outcomes
☒	☐	Estimates of effect sizes (e.g. Cohen's d, Pearson's r), indicating how they were calculated

Our web collection on statistics for biologists contains articles on many of the points above.

Software and code

Policy information about availability of computer code

Data collection	Flow cytometry data were collected by BD LSR Fortessa and BD FACSDiva (v8.0.2). RNA-Seq was sequenced using an Illumina NovaSeq 6000 (PE150) platform. Illumina Nova6000 sequencer was used to check the CUT&Tag sequencing read quality. qPCR was performed by the ABI prism 7500 real-time PCR System (Thermo Fisher). The HRP signal of western blotting was developed by electrochemiluminescence (ChemeMINI610) and collected by Sage Capture (v2.19.12). The electron microscopy data was collected by using Hitachi HT-7800 TEM system (v01.20) and option camera of AMT-XR81DIR. Quantitication of cellular metabolites were analyzed by UHPLC-MS/MS and Compound Discoverer (v3.1). Stable isotope labelling experiments were performed by GC-HRMS and UPLC-TQMS. The metabolites were identified and quantified by Xcalibur (v4.1) and TraceFinder (v5.1).Seahorse was performed by seahorse XF24 analyzer (Agilent).
Data analysis	Flow cytometry data were collected by FlowJo (v10.4). Statistics and Data plotting were performed by GraphPad Prism (v8.0.1). RNA-seq raw read counts were normalized by their library size factors using DESeq2 (v1.28.1). The GO and KEGG pathway enrichment analysis was performed with cluster Profiler (v3.16.0). Expression heat maps were generated with the R package 'pheatmap' (v1.0.12). GSEA (v.4.0) was used for GSEA analysis. FastQC (v0.11.4) was used to analyze the CUT&Tag sequencing read quality. Reads were quality trimmed to a minimum phred score of 20 using trimmomatic (v0.39). All reads produced by CUT&Tag-seq were aligned to the hg38 human genome using Bowtie2 (v2.2.8). Peaks were called using MACS2 (v2.2.6) and annotated with ChIPseeker (v1.22.1). The visualizations were created using deepTools (v3.5.1) and pyGenomeTracks (v3.7). Western blotting analysis was performed using Image J (v1.8.0) software. Metaboanalyst (v5.0)software was used for metabolomic data analysis. MassLynx software (v4.1, Waters) and R package (v4.1.1) were used for statistical analysis of 13C-labeling metabolites. OCR and ECAR were analyzed by seahorse wave software (Seahorse, Agilent Technologies. v2.6).

For manuscripts utilizing custom algorithms or software that are central to the research but not yet described in published literature, software must be made available to editors and reviewers. We strongly encourage code deposition in a community repository (e.g. GitHub). See the Nature Portfolio guidelines for submitting code & software for further information.

Data

Policy information about availability of data

All manuscripts must include a data availability statement. This statement should provide the following information, where applicable:

- Accession codes, unique identifiers, or web links for publicly available datasets
- A description of any restrictions on data availability
- For clinical datasets or third party data, please ensure that the statement adheres to our policy

The RNA-seq raw datasets generated during this study are deposited to NCBI GEO database with the accession number GSE216623 and GSE219257. The CUT&Tag-seq datasets are available at GEO (accession number GSE216623). Metabolomics data have been deposited to the EMBL-EBI MetaboLights database with the identifier MTBLS6661 (https://www.ebi.ac.uk/metabolights/MTBLS6661). Any additional materials and reagents are available from the corresponding author upon reasonable request. Source data are provided with this paper.

Human research participants

Policy information about studies involving human research participants and Sex and Gender in Research.

Reporting on sex and gender	*Use the terms sex (biological attribute) and gender (shaped by social and cultural circumstances) carefully in order to avoid confusing both terms. Indicate if findings apply to only one sex or gender; describe whether sex and gender were considered in study design whether sex and/or gender was determined based on self-reporting or assigned and methods used. Provide in the source data disaggregated sex and gender data where this information has been collected, and consent has been obtained for sharing of individual-level data; provide overall numbers in this Reporting Summary. Please state if this information has not been collected. Report sex- and gender-based analyses where performed, justify reasons for lack of sex- and gender-based analysis.*
Population characteristics	*Describe the covariate-relevant population characteristics of the human research participants (e.g. age, genotypic information, past and current diagnosis and treatment categories). If you filled out the behavioural & social sciences study design questions and have nothing to add here, write "See above."*
Recruitment	*Describe how participants were recruited. Outline any potential self-selection bias or other biases that may be present and how these are likely to impact results.*
Ethics oversight	*Identify the organization(s) that approved the study protocol.*

Note that full information on the approval of the study protocol must also be provided in the manuscript.

Field-specific reporting

Please select the one below that is the best fit for your research. If you are not sure, read the appropriate sections before making your selection.

☒ Life sciences ☐ Behavioural & social sciences ☐ Ecological, evolutionary & environmental sciences

For a reference copy of the document with all sections, see nature.com/documents/nr-reporting-summary-flat.pdf

Life sciences study design

All studies must disclose on these points even when the disclosure is negative.

Sample size	The sample size for each experiment is indicated in the figures and figure legends. Sample sizes were based on our experience and common practice in the related fields, balancing statistic robustness, resource availability and animal welfare. No Statistical methods were used to predetermine sample size. For in vitro experiments, at least three independent sample were achieved. n= 4 samples were used for evaluation of the levels of metabolites in cells (ref. 48,50). n= 3-4 samples were used for RNA-seq (ref. 48,50) and n= 2 samples were used for CUT&Tag-seq (ref. 75). n=3-4 samples were used for isotope tracing assay (ref. 60,78,79). For in vivo experiments, n = 4-8 mice were used per experimental group. n=40-50 cells were used for statistics of mitochondrial area (ref. 26,50).
Data exclusions	No data was excluded from this study.
Replication	Biological replicates are included to ensure the reproducibility and all repeated experiments are successful. For ex vivo experiments, the number of replicates is equal to individual mice used and are independently repeated as reported. For in vivo studies, at least five mice were used for each single groups and all experiments are independently repeated at least twice with similar results to make the conclusion. For other experiments, at least two biological replicates were included and performed independently to ensure the reproducibility. When representative data are shown, the experimental findings were reproduced independently with similar results.
Randomization	Randomisation was applied wherever possible. For example, the samples of RNA-seq , Metabolomic, isotope tracing and CUT&Tag-seq were processed and subjected to the platform in random orders. For animal experiments, Mice are sex- and age- matched and are randomly assigned to different treatment and control groups. Otherwise, randomisation was not performed. For example, when performing

	immunoblotting, samples needed to be loaded in a specific order to generate the final figures. Before adoptive transfer of T cells, the tumor-bearing mice were equally allocated into different treatment and control groups according to the tumor size.
Blinding	For the in vivo tumor experiments, all the mice were coded and the data collection such as tumor measuring and mice survival recording was performed by researcher without the knowledge of the codes. Data analysis was performed by researchers blinded to group allocation. No blinding was involved in other experiments, as machine-based readouts are not subject to investigator bias.

Reporting for specific materials, systems and methods

We require information from authors about some types of materials, experimental systems and methods used in many studies. Here, indicate whether each material, system or method listed is relevant to your study. If you are not sure if a list item applies to your research, read the appropriate section before selecting a response.

Materials & experimental systems

n/a	Involved in the study
☐	☒ Antibodies
☐	☒ Eukaryotic cell lines
☒	☐ Palaeontology and archaeology
☐	☒ Animals and other organisms
☒	☐ Clinical data
☒	☐ Dual use research of concern

Methods

n/a	Involved in the study
☐	☒ ChIP-seq
☐	☒ Flow cytometry
☒	☐ MRI-based neuroimaging

Antibodies

Antibodies used	Antibodies used for flow cytometric analysis: APC anti-human NGFR (cat:345108, 1:1,000 for FACS), FITC anti-human CD4 (cat:357406, 1:200 for FACS), Pacific Blue anti-human CD8α (cat:300928, 1:200 for FACS), APC-Cy7 anti-human/mouse CD44 (cat:103028, 1:200 for FACS), PE anti-human CCR7 (cat:353204, 1:200 for FACS), PE anti-human TNF-α (cat:502909, 1:200 for FACS), PE-Cy7 anti-human CD45RO (cat:304230, 1:200 for FACS), APC anti-human IFN-γ (cat:502512, 1:200 for FACS), PE-Cy7 anti-human LAG-3 (cat:369310, 1:200 for FACS), PE anti-human TIM-3 (cat:345006, 1:200 for FACS), BV711 anti-human PD-1 (cat:329928, 1:200 for FACS), Percp-Cy5.5 anti-human CD62L (cat:304824, 1:200 for FACS), APC anti-human CD27 (cat:302810, 1:200 for FACS), BV711 anti-mouse CD8α (cat:100748, 1:200 for FACS), PE-Cy7 anti-mouse CD62L (cat:104418, 1:200 for FACS), APC-Cy7 anti-mouse CD45.2 (cat:830789, 1:200 for FACS), Pacific Blue anti-mouse CD45.1 (cat:110722, 1:200 for FACS), APC anti-mouse Ly108 (cat:134610, 1:200 for FACS), PE anti-mouse LAG-3 (cat:125207, 1:200 for FACS), Alexa Fluor 488 anti-c-MYC Antibody (cat:626811, 1:200 for FACS), PE anti-mouse TNF-α (cat:506306, 1:200 for FACS) and APC anti-mouse IFN-γ (cat:505810, 1:200 for FACS) were purchased from BioLegend. Live/Dead Fixable Dead Cell stain kit, Percp-efluor710 anti-mouse PD-1 (cat:46-9981-82, 1:200 for FACS), PE Phospho-S6 (Ser235/236) (cat:12-9007-42, 1:200 for FACS), PE anti-human/mouse TOX (cat:12-6502-82, 1:200 for FACS), and PE-Cy7 anti-mouse TIM-3 (cat:25-5870-82, 1:200 for FACS) were purchased from Thermo Fisher. Alexa Fluor647 anti-TCF1 (cat:6709, 1:200 for FACS) and Phospho-4E-BP1 (Thr37/46) (cat:2846, 1:200 for FACS) were purchased from Cell Signaling Technology. Alexa Fluor 647 anti-Puromycin (cat:MABE343-AF647, 1:800 for FACS) was purchased from Merck.<br>Antibodies used for WB: Rabbit anti-phospho-Akt (Ser473) (cat:#4060, 1:1,000 for IB), anti-phospho-NF-κB p65 (Ser536) (cat:#3033, 1:1,000 for IB), anti-c-MYC (cat:#18583, 1:1,000 for IB), anti-EZH2 (cat:#5246, 1:1,000 for IB), anti-Tri-Methyl-Histone H3 (Lys27) (cat:#9733, 1:1,000 for IB), anti-Tri-Methyl-Histone H3 (Lys4) (cat:#9751, 1:1,000 for IB), anti-Di-Methyl-Histone H3 (Lys9) (cat:#4658, 1:1,000 for IB), anti-Di-Methyl-Histone H3 (Lys79) (cat:#5427, 1:1,000 for IB), anti-Histone H3 (cat:#9715, 1:1,000 for IB), anti-COXIV (cat:#4850, 1:1,000 for IB), anti-rabbit IgG (HRP-linked) (cat:#7074, 1:2,000 for IB), and anti-mouse IgG (HRP-linked) (cat:#7076, 1:2,000 for IB) were purchased from Cell Signaling Technology. Mouse anti-Tim23 (cat:611223, 1:1,000 for IB) was from BD Biosciences. Mouse anti-β-Actin (cat:66009-1-Ig, 1:5,000 for IB), anti-SLC7A5 (cat:67951-1-Ig, 1:1,000 for IB), and Rabbit anti-SLC38A1 (cat:12039-1-AP, 1:1,000 for IB) were from Proteintech. Rabbit anti-SLC38A2 (cat:BMP081, 1:1,000 for IB) was purchased from MBL (MEDICAL & BIOLOHICAL LABORATORIES CO., LTD).
Validation	The following commercially available antibodies were validated by the company, as well as other researchers (as the information collected by the RRID database):<br>APC anti-human NGFR (cat:345108, RRID:AB_10645515), FITC anti-human CD4 (cat:357406, RRID:AB_2562357), Pacific Blue anti-human CD8α (cat:300928, RRID:AB_10612929), APC-Cy7 anti-human/mouse CD44 (cat:103028, RRID:AB_830785), PE anti-human CCR7 (cat:353204, RRID:AB_10913813), PE anti-human TNF-α (cat:502909, RRID:AB_315261), PE-Cy7 anti-human CD45RO (cat:304230, RRID:AB_11203900), APC anti-human IFN-γ (cat:502512, RRID:AB_315237), PE-Cy7 anti-human LAG-3 (cat:369310, RRID:AB_2629753), PE anti-human TIM-3 (cat:345006, RRID:AB_2116576), BV711 anti-human PD-1 (cat:329928, RRID:AB_2562911), Percp-Cy5.5 anti-human CD62L (cat:304824, RRID:AB_2239105), APC anti-human CD27 (cat:302810, RRID:AB_314302), BV711 anti-mouse CD8α (cat:100748, RRID:AB_2562100), PE-Cy7 anti-mouse CD62L (cat:104418, RRID:AB_313103), APC-Cy7 anti-mouse CD45.2 (cat:109824, RRID:AB_830789), Pacific Blue anti-mouse CD45.1 (cat:110722, RRID:AB_492866), APC anti-mouse Ly108 (cat:134610, RRID:AB_2728155), PE anti-mouse LAG-3 (cat:125207, RRID:AB_2133344), Alexa Fluor 488 anti-c-MYC Antibody (cat:626811, RRID:AB_2888733), PE anti-mouse TNF-α (cat:506306, RRID:AB_315427), and APC anti-mouse IFN-γ (cat:505810, RRID:AB_315404) were purchased from BioLegend. Percp-efluor710 anti-mouse PD-1 (cat:46-9981-82, RRID:AB_11151142), PE-Cy7 anti-mouse TIM-3 (cat:25-5870-82, RRID:AB_2573483), PE Phospho-S6 (Ser235/236) (cat:12-9007-42, RRID:AB_2572667), and PE anti-human/mouse TOX (cat:12-6502-82, RRID:AB_10855034) were purchased from Thermo Fisher. Alexa Fluor 647 anti-TCF1 (cat:6709, RRID:AB_2797631) and Phospho-4E-BP1 (Thr37/46) (cat:2846S, RRID:AB_2292749) were purchased from Cell Signaling Technology. Alexa Fluor 647 anti-Puromycin (cat:MABE343-AF647, RRID:AB_2736876) was purchased from Merck. Rabbit anti-phospho-Akt

(Ser473) (cat#4060, RRID:AB_2315049), anti-phospho-NF-κB p65 (Ser536) (cat#3033, RRID:AB_331284), anti-c-MYC (cat#18583, RRID:AB_2895543), anti-EZH2 (cat#5246, RRID:AB_10694683), anti-Tri-Methyl-Histone H3 (Lys27) (cat#9733, RRID:AB_2616029), anti-Tri-Methyl-Histone H3 (Lys4) (cat#9751, RRID:AB_2616028), anti-Di-Methyl-Histone H3 (Lys9) (cat#4658, RRID:AB_10544405), anti-Di-Methyl-Histone H3 (Lys79) (cat#5427, RRID:AB_10693787), anti-Histone H3 (cat#9715, RRID:AB_331563), anti-COXIV (cat#4850, RRID:AB_2085424), anti-rabbit IgG (HRP-linked) (cat#7074, RRID:AB_954861), and anti-mouse IgG (HRP-linked) (cat#7076, RRID:AB_330924) were purchased from Cell Signaling Technology. Mouse anti-Tim23 (cat:611223, RRID:AB_398755) was from BD Biosciences. Mouse anti-β-Actin (cat:66009-1-Ig, RRID:AB_2687938), anti-SLC7A5 (cat:67951-1-Ig, RRID:AB_2918703), and Rabbit anti-SLC38A1 (cat:12039-1-AP, RRID:AB_2190392) were from Proteintech. Rabbit anti-SLC38A2 (cat:BMP081, RRID:AB_10597880) was purchased from MBL (MEDICAL & BIOLOHICAL LABORATORIES CO., LTD).

Eukaryotic cell lines

Policy information about cell lines and Sex and Gender in Research

Cell line source(s)

HEK 293T cells from American Type Culture Collection (ATCC) were used to produce virus in this study. The mouse melanoma cell line, B16(H-2Kb), from ATCC was transduced to express OVA peptide. K562 cells from ATCC were transduced to express human CD19 and cultured in RPMI 1640 medium. Human peripheral blood mononuclear cells(PBMC) from healthy donors were purchased from Sailybio (Shanghai, China).

Authentication

HEK 293T, B16(H-2Kb), and K562 cells were obtained from and pre-authenticated by ATCC by STR sequencing. Human PBMCs were obtained from Sailybio and confirmed by morphology and FACS.

Mycoplasma contamination

All cell lines were free of mycoplasma contamination.

Commonly misidentified lines
(See ICLAC register)

No commonly misidentified cell lines were used in the study.

Animals and other research organisms

Policy information about studies involving animals; ARRIVE guidelines recommended for reporting animal research, and Sex and Gender in Research

Laboratory animals

CD45.1+ OT-I TCR transgenic mice on a C57BL/6N background were housed. CD45.2+ female C57BL/6N mice were purchased from the Vital River Co, Ltd (Beijing, China) as recipients. For one independent experiment in vivo, the CD45.1+ and CD45.2+ mice (6–12 weeks) used were sex-matched. Female NCG (NOD/ShiLtJGpt-Prkdcem26Cd52Il2rgem26Cd22/Gpt) mice (6–12 weeks) were purchased from GemPharmatech (Nanjing, China). Mice were housed on a standard condition, with 12 h/12 h light/dark cycles, controlled temperature of 22–24°C and humidity of 60%, with unrestricted food and water availability and examined daily.

Wild animals

No wild animal were used in this study.

Reporting on sex

In our study, we used female and male CD45.1+ OT-I TCR transgenic mice in vitro experiments. Our conclusions from experiments in vitro were not affected by sex of mice. The ACT experiments in vivo were used female mice, and thus could not confirm the influence of sex.

Field-collected samples

No field-collected samples were used in the studies

Ethics oversight

All animal experiments were performed with the approval of the Institutional Animal Care and Use Committee (IACUC) of Suzhou Institute of Systems Medicine (ISM-IACUC-0151-R).

Note that full information on the approval of the study protocol must also be provided in the manuscript.

ChIP-seq

Data deposition

☒ Confirm that both raw and final processed data have been deposited in a public database such as GEO.

☒ Confirm that you have deposited or provided access to graph files (e.g. BED files) for the called peaks.

Data access links
May remain private before publication.

The CUT&Tag-seq datasets are available at GEO (accession number GSE216623).

Files in database submission

H3K27me3_10mM_MET_1_1.fq.gz
H3K27me3_10mM_MET_1_2.fq.gz
H3K27me3_10mM_MET_2_1.fq.gz
H3K27me3_10mM_MET_2_2.fq.gz
H3K27me3_10mM_1_1.fq.gz
H3K27me3_10mM_1_2.fq.gz
H3K27me3_10mM_2_1.fq.gz
H3K27me3_10mM_2_2.fq.gz
H3K27me3_CON_1_1.fq.gz

H3K27me3_CON_1_2.fq.gz
H3K27me3_CON_2_1.fq.gz
H3K27me3_CON_2_2.fq.gz
H3K27me3_10mM_MET_1.bigwig
H3K27me3_10mM_MET_2.bigwig
H3K27me3_10mM_1.bigwig
H3K27me3_10mM_2.bigwig
H3K27me3_CON_1.bigwig
H3K27me3_CON_2.bigwig

H3K4me3_10mM_MET_1_1.fq.gz
H3K4me3_10mM_MET_1_2.fq.gz
H3K4me3_10mM_MET_2_1.fq.gz
H3K4me3_10mM_MET_2_2.fq.gz
H3K4me3_10mM_1_1.fq.gz
H3K4me3_10mM_1_2.fq.gz
H3K4me3_10mM_2_1.fq.gz
H3K4me3_10mM_2_2.fq.gz
H3K4me3_CON_1_1.fq.gz
H3K4me3_CON_1_2.fq.gz
H3K4me3_CON_2_1.fq.gz
H3K4me3_CON_2_2.fq.gz
H3K4me3_10mM_MET_1.bigwig
H3K4me3_10mM_MET_2.bigwig
H3K4me3_10mM_1.bigwig
H3K4me3_10mM_2.bigwig
H3K4me3_CON_1.bigwig
H3K4me3_CON_2.bigwig

Genome browser session
(e.g. UCSC)

http://genome.ucsc.edu/cgi-bin/hgGateway

Methodology

Replicates

CUT&Tag ChIP-seq data are from two independent replicates.

Sequencing depth

Paired-end 150 sequencing was performed on an Illumina Nova 6000 and 18 to 25 million reads were generated for each sample.

Antibodies

Tri-Methyl-Histone H3 (Lys27) (C36B11) Rabbit mAb 1:100
Tri-Methyl-Histone H3 (Lys4) (C42D8) Rabbit mAb 1:100

Peak calling parameters

H3K27me3 peaks were called using MACS2 (v2.2.6) with options: --broad --broad-cutoff 0.1;
H3K4me3 peaks were called using MACS2 (v2.2.6) with options: -q 0.01;

Data quality

FastQC (v0.11.4) was used to check the sequencing read quality

Number of peaks that called by MACS2 (H3K27me3 : FDR < 5%, fold enrichment >= 3; H3K4me3 : FDR < 5%, fold enrichment >= 5),

18469 H3K27me3_10mM_MET_1
13984 H3K27me3_10mM_MET_2
15479 H3K27me3_10mM_1
17844 H3K27me3_10mM_2
20656 H3K27me3_CON_1
14147 H3K27me3_CON_1

12402 H3K4me3_10mM_MET_1
11788 H3K4me3_10mM_MET_2
11557 H3K4me3_10mM_1
12352 H3K4me3_10mM_2
12500 H3K4me3_CON_1
12321 H3K4me3_CON_1

Software

All raw sequence data were quality trimmed to a minimum phred score of 20 using trimmomatic (v0.39).
For standardization between experiments, the ChIPseqSpikeInFree method was used to normalize sample without spike-in DNA based on the recommendation of CUT&Tag protocol. Reads were aligned using Bowtie2 (v2.2.8) with options: --local --very-sensitive-

local --no-unal --no-mixed --no-discordant --phred33 -I 10 -X 700. Peaks were annotated with ChIPseeker (v1.22.1), and visualizations were created using deepTools (v3.5.1) and pyGenomeTracks (v3.7).

Flow Cytometry

Plots

Confirm that:

- [x] The axis labels state the marker and fluorochrome used (e.g. CD4-FITC).
- [x] The axis scales are clearly visible. Include numbers along axes only for bottom left plot of group (a 'group' is an analysis of identical markers).
- [x] All plots are contour plots with outliers or pseudocolor plots.
- [x] A numerical value for number of cells or percentage (with statistics) is provided.

Methodology

Sample preparation	Mouse CD8+ T lymphocytes were isolated from the spleens of 6-8-week OT-1 mice using Nylon Wool Fiber（Seebio）and cultured in RPMI-1640 medium supplemented with 10% FBS, 1% Penicillin-Streptomycin (PS), 1% non-essential amino acids, 1% GlutaMAX, 1mM sodium pyruvate, 0.1M HEPES and 50μM β-mercaptoethanol in the presence of mouse IL-2 (20 U/ml, Peprotech). Purified cells were activated by using plate-bound anti-mouse CD3 (2μg/ml, Biolegend) plus soluble anti-mouse CD28 (1μg/ml, Biolegend) monoclonal antibodies for 24 h. Peripheral blood mononuclear cells (PBMC) were cultured in RMPI-1640 medium supplemented with 5% Human Serum AB(Gemini), 1% PS, 1% non-essential amino acids, 1% GlutaMAX, 1mM sodium pyruvate, 0.1M HEPES, 50μM β-mercaptoethanol in the presence of human IL-2 (100U/ml, Peprotech). Plate-bound anti-human CD3 (1μg/ml, Biolegend) and soluble anti-human CD28 (1μg/ml, Biolegend) monoclonal antibodies were used to activate naïve T cells for three days.<br>Spleen, lymph nodes and tumor were passed through 70 micron filters.<br>Cells were stained with fluorescent antibodies and then analyzed by flow cytometry. For surface marker staining, cells were stained with fluorescently conjugated antibodies and Live/Dead Fixable Dead Cell stain kit (Invitrogen) in FACS buffer (phosphate-buffered saline (PBS) with 2% FBS), then fixed with 2% paraformaldehyde (Casmart) for 20min at room temperature. For intracellular staining of phospho-proteins, pre-stained cells were fixed with Fixation Buffer (BioLegend) and then stained with phospho-specific antibodies in Permeabilization Buffer (Invitrogen). For detection of intracellular cytokines, cells were stimulated with phorbol myristate acetate (PMA) in presence of Brefeldin A (BFA) (BioLegend) for 4.5 h. Then, the pre-stained cells were fixed and stained with cytokines antibodies in Permeabilization Buffer. For intracellular transcriptional factors staining, cells were pre-stained with Live/Dead Fixable Dead Cell stain kit and fluorescent conjugated antibodies in FACS buffer for surface markers. The cells were then fixed for 30 min on ice using FOXP3/Transcription Factor Fixation Buffer (Invitrogen) and stained with transcription factor antibodies in Permeabilization Buffer. After staining, cells were resuspended in FACS buffer for flow cytometry.
Instrument	LSR Fortessa (BD Biosciences)
Software	BD FACSDiva (v8.0.2) was used to collect data. FlowJo (v10.4) was used to analyze the flow cytometry data.
Cell population abundance	Expanded CD8+ OT-1 T cell abundance is higher than 90% with flow cytometry validation.
Gating strategy	Cells were identified with FSC-A/SSC-A gating and followed by SSC-H/SSC-A for single cells. Live cells were distinguished based on the staining of Live/Dead. For analysis of T cell infiltration in tumors, gating strategy is shown in Extended Data Fig. 10B.

- [x] Tick this box to confirm that a figure exemplifying the gating strategy is provided in the Supplementary Information.
