# Supplementary material for: Extracellular acidosis restricts one-carbon metabolism and preserves T cell stemness
Source: Nat Metab. 2023 Jan 30;5(2):314–30. doi: 10.1038/s42255-022-00730-6 (PMC9970874; doi:10.1038/s42255-022-00730-6)

Fig.3F

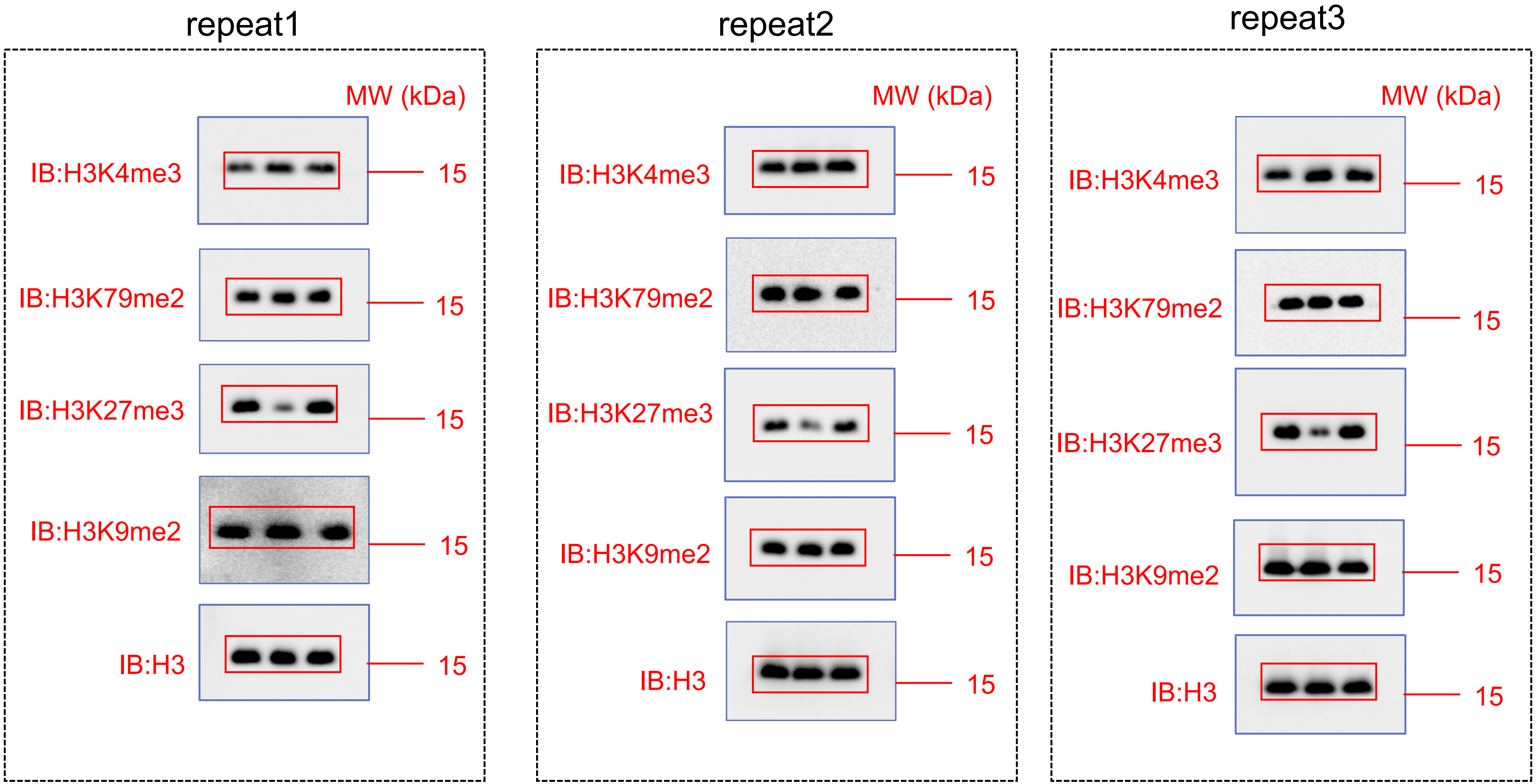
repeat1
MW (kDa)
IB:H3K4me3
15
IB:H3K79me2
15
IB:H3K27me3
15
IB:H3K9me2
15
IB:H3
15
repeat2
MW (kDa)
IB:H3K4me3
15
IB:H3K79me2
15
IB:H3K27me3
15
IB:H3K9me2
15
IB:H3
15
repeat3
MW (kDa)
IB:H3K4me3
15
IB:H3K79me2
15
IB:H3K27me3
15
IB:H3K9me2
15
IB:H3
15

Fig.4I

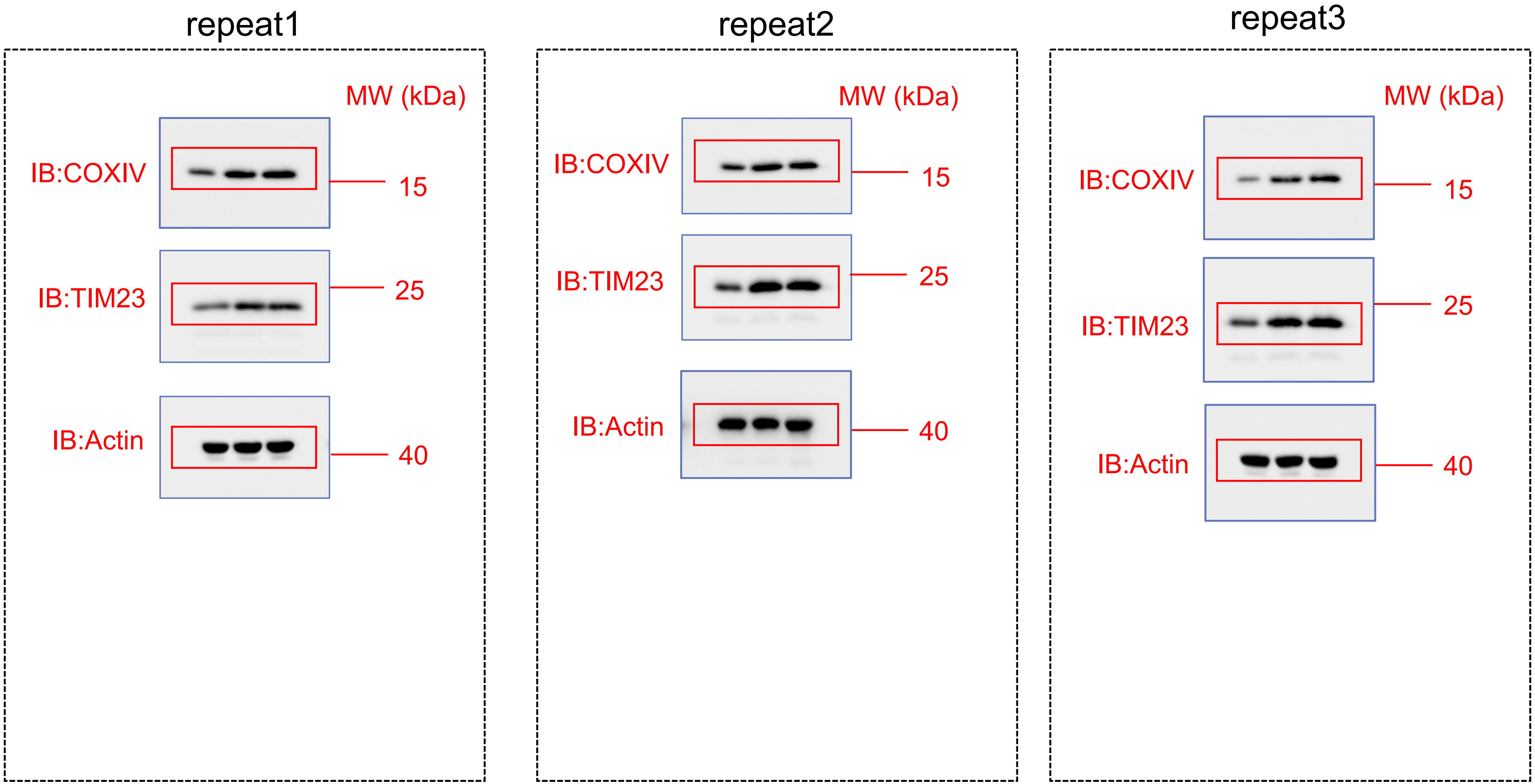
repeat1
MW (kDa)
IB:COXIV
15
IB:TIM23
25
IB:Actin
40
repeat2
MW (kDa)
IB:COXIV
15
IB:TIM23
25
IB:Actin
40
repeat3
MW (kDa)
IB:COXIV
15
IB:TIM23
25
IB:Actin
40

Extended Data Fig.3C

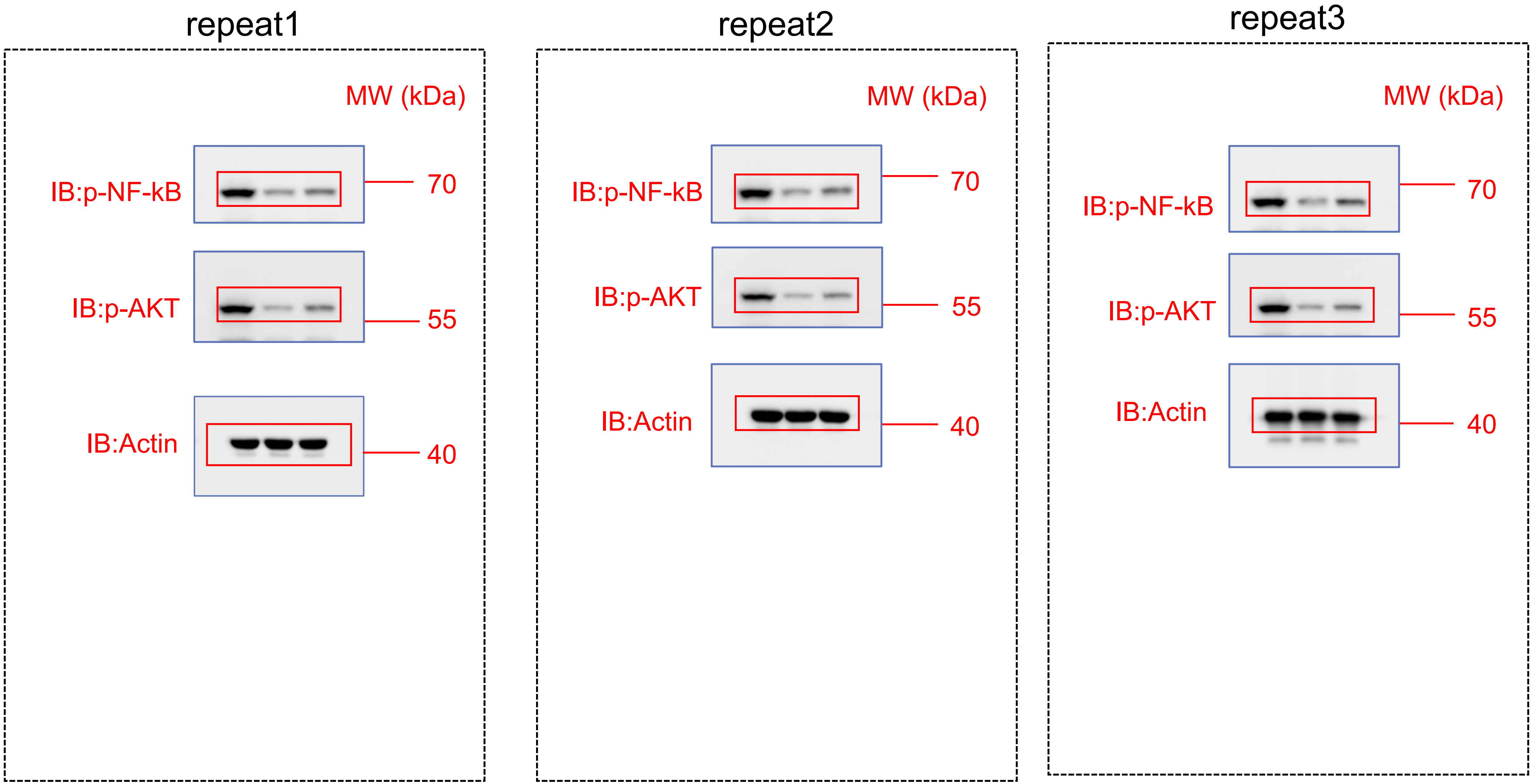

Extended Data Fig.5D

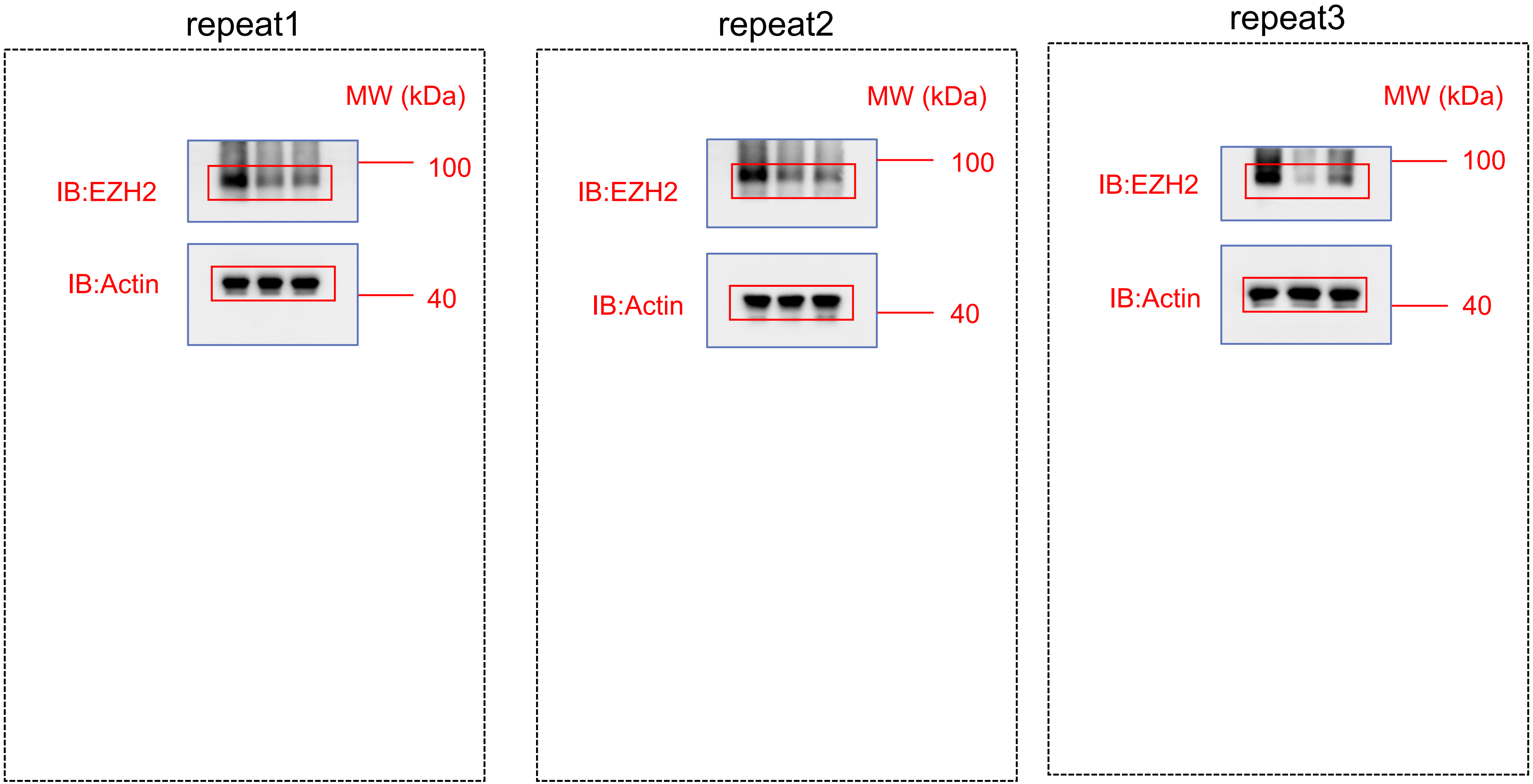

Extended Data Fig.6B

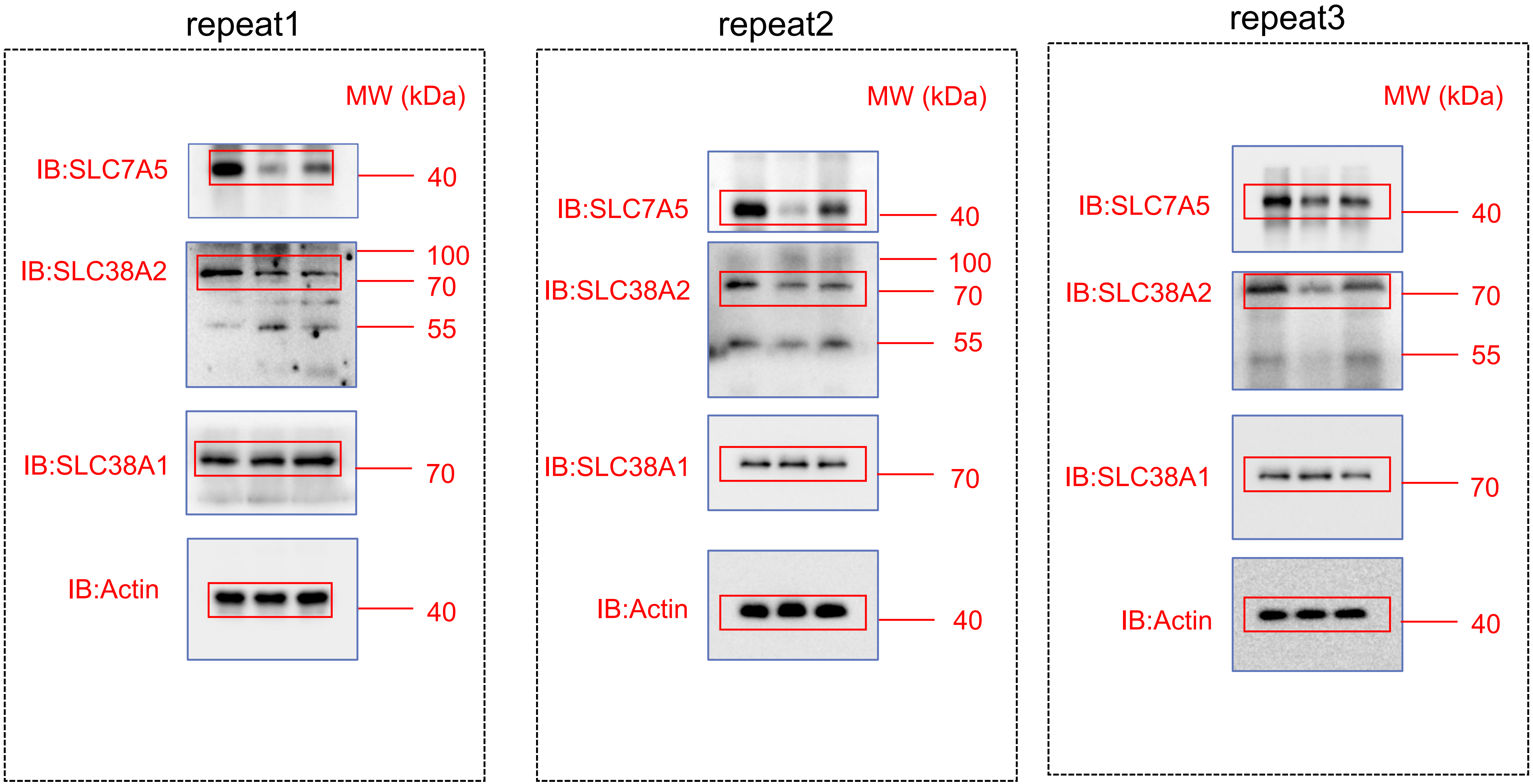

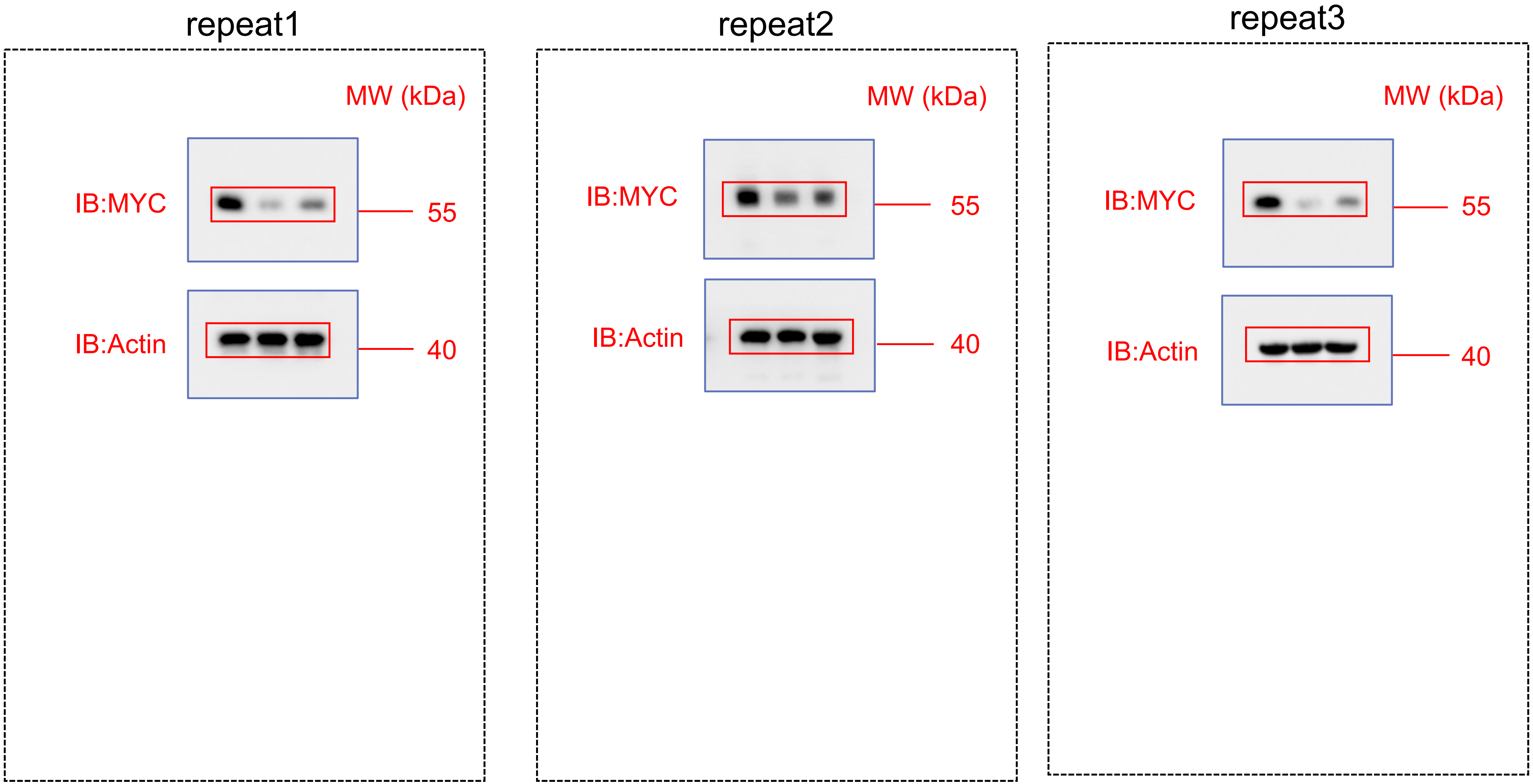

Extended Data Fig.6D

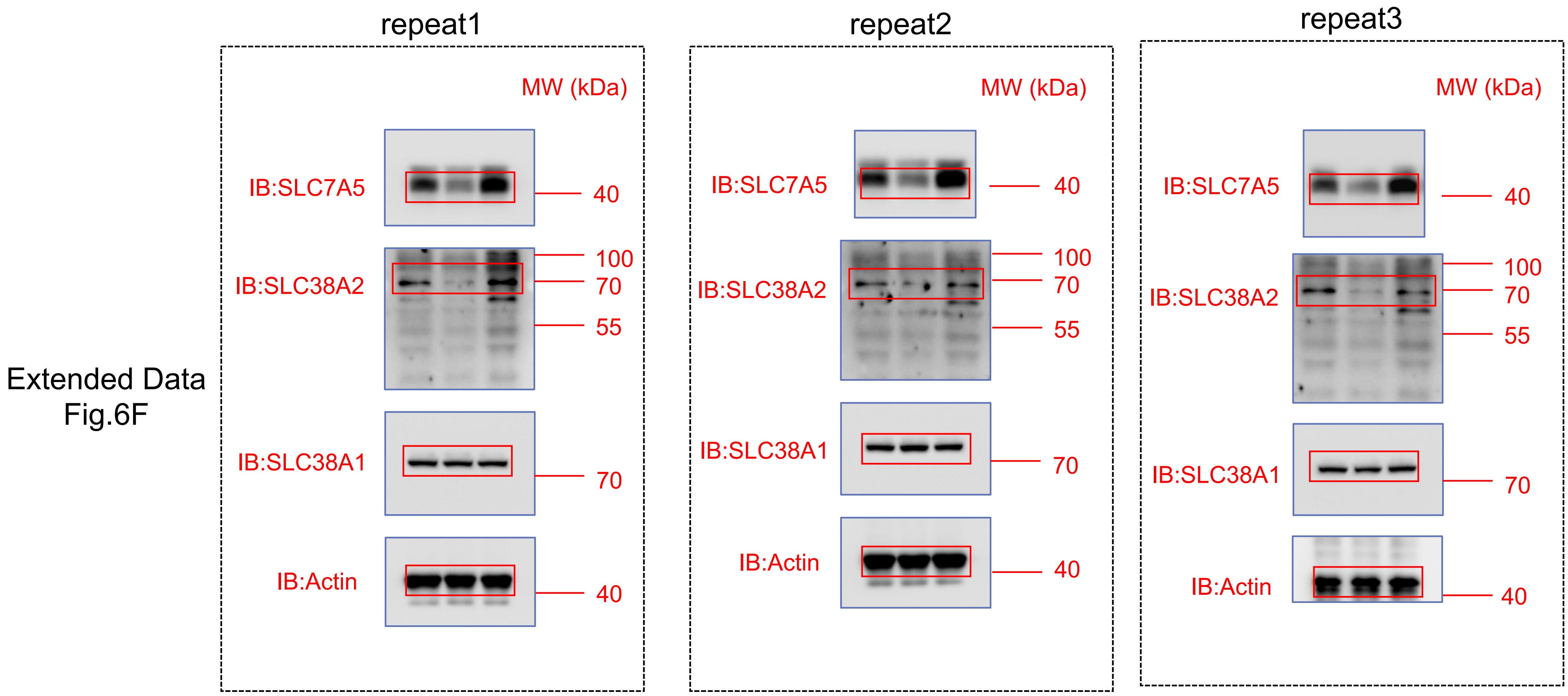
Extended Data
Fig.6F
repeat1
MW (kDa)
IB:SLC7A5
40
IB:SLC38A2
100
70
55
IB:SLC38A1
70
IB:Actin
40
repeat2
MW (kDa)
IB:SLC7A5
40
IB:SLC38A2
100
70
55
IB:SLC38A1
70
IB:Actin
40
repeat3
MW (kDa)
IB:SLC7A5
40
IB:SLC38A2
100
70
55
IB:SLC38A1
70
IB:Actin
40

Extended Data Fig.6G

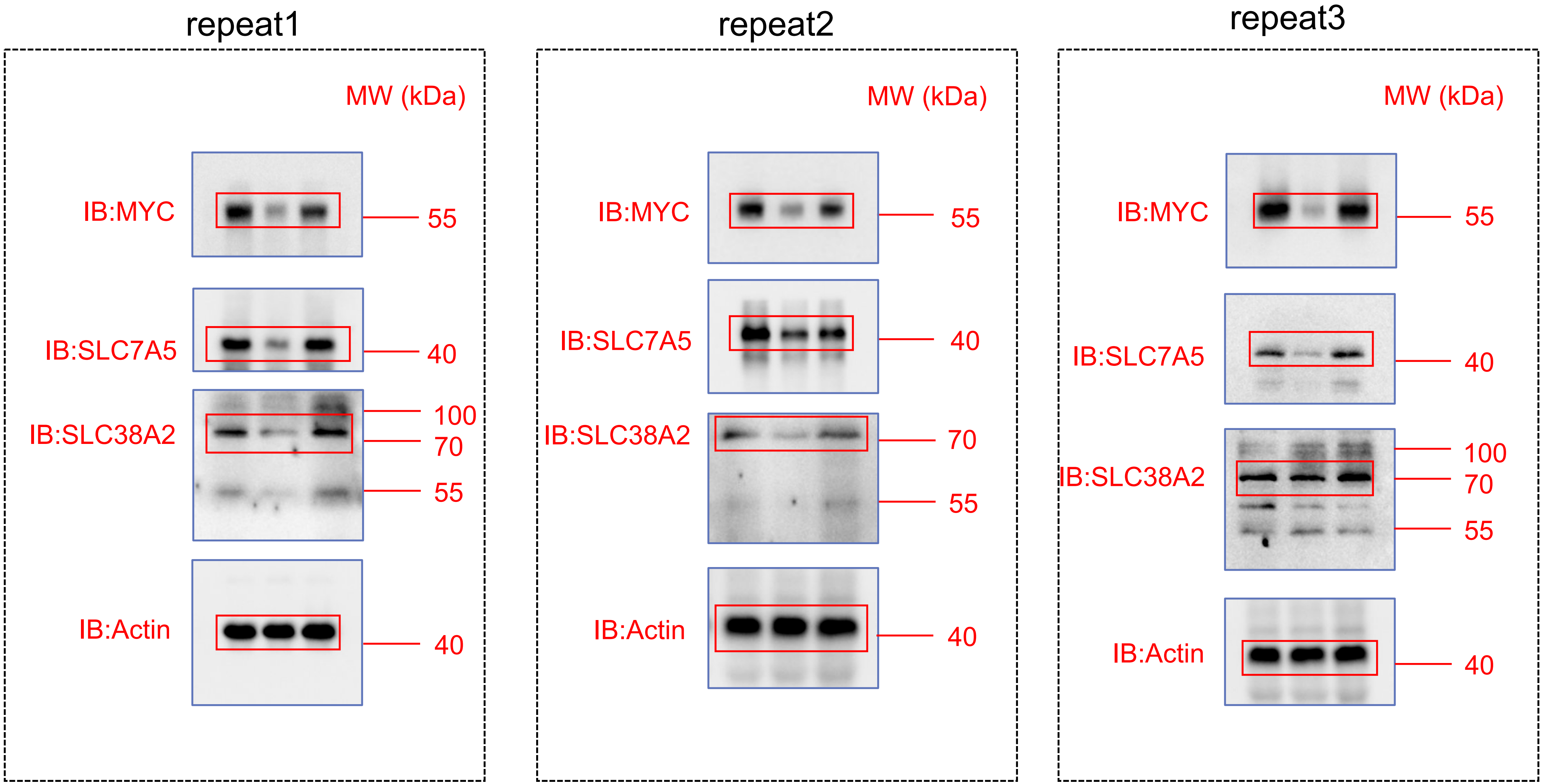

Supplement: Source data unprocessed western blots — Unprocessed western blots [file 42255_2022_730_MOESM20_ESM.pdf]
